# Biodegradable Aurum: Gold Nanosheets Undergo Biodegradation by Neutrophil Myeloperoxidase

**DOI:** 10.1002/smsc.202500491

**Published:** 2026-04-24

**Authors:** Pavithra Kurungottu, Aravind Kannoth Anilkumar, Soumyadeep Poddar, K. Swetha, Parvathy Anil, Srinivasa Reddy Bonam, Rajendra Kurapati

**Affiliations:** ^1^ School of Chemistry Indian Institute of Science Education and Research Maruthamala PO Vithura Thiruvananthapuram 695551 India; ^2^ Vaccine Immunology Laboratory Department of Applied Biology CSIR‐Indian Institute of Chemical Technology Hyderabad 500007 India; ^3^ Academy of Scientific and Innovative Research Ghaziabad 201002 India

**Keywords:** biodegradation, gold, gold nanosheet, myeloperoxidase, nanomaterials, neutrophils, photothermal therapy

## Abstract

Though gold nanomaterials (NMs) have been largely used in biomedical applications for decades, none have been approved for clinical usage, mostly due to a lack of understanding about their long‐term fate and non‐biodegradability. Here, the biodegradability of 2D Au nanosheets (AuNS) by enzymatic catalysis of human myeloperoxidase (hMPO) using a test‐tube model and an in vitro model with MPO‐secreting neutrophil‐like cells (present in the blood) differentiated from human leukemia (HL‐60) cells has been reported. The results obtained from the high‐resolution transmission electron microscopy (HR‐TEM), selected area diffraction (SAED), X‐ray photoelectron spectroscopy (XPS), and electrical impedance spectroscopy (EIS) confirm that the AuNS undergoes partial biodegradation to Au^I^ ions by reactive radical intermediates generated by hMPO, hypochlorous acid, including hydroxy radicals generated by AuNS. The results demonstrate the potential for AuNS biodegradation by neutrophils, which are primarily present in the blood. Therefore, these results can be crucial for understanding the long‐term fate of AuNMs in humans. Further, near‐infrared (NIR) photothermal therapy is successfully demonstrated using AuNS against triple‐negative breast cancer cells.

## Graphical abstract

The gold nanosheets undergo biodegradation when interacting with human neutrophil‐like cells that secrete myeloperoxidase (MPO). The gold sheets are degraded by reactive radical enzyme intermediates of MPO, HOCl and hydroxy radicals generated via Fenton‐like reaction. These results suggest the possible biodegradation of gold nanomaterials in the blood.

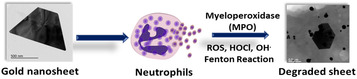

## 1 Introduction

Gold was used in medicine in ancient times. Though many biomedical applications of gold nanomaterials (AuNMs) in drug delivery, biomedical imaging, photothermal therapies, and vaccine adjuvants have been reported, none have been tested in clinical trials.^[^ ^1^, ^2^ ^]^ Indeed, the clinical translation of nanomaterials (NMs) is mainly dependent upon their long‐term in vivo biocompatibility (toxicity, distribution) and biodegradation.^[^ ^3^ ^]^ The biocompatibility of NMs can be modulated by surface functionalization with proteins, biopolymers, and other biomolecules, while biodegradability is an inherent property.^[^ ^4^ ^]^ The in vivo biocompatibility studies revealed the distribution of AuNMs in the liver and spleen.^[^ ^5^, ^6^, ^7^ ^]^ Only in 2020, Florent Carn and Florence Gazeau reported the unexpected biotransformation of Au nanoparticles (AuNPs, 4–22 nm) in the primary fibroblasts up to 6 months, where AuNPs underwent oxidation to Au^I^ ions inside the lysosome by reactive oxygen species (ROS) generated via NADPH oxidase (NOX proteins) and the resulting Au^I^ ions were recrystallized to form aurosome (gold salts).^[^ ^8^ ^]^ These results revealed that intracellular biotransformation of AuNPs is possible, which could impact the long‐term toxicity and in vivo clearance of AuNMs. Therefore, further studies are needed to understand the potential biodegradation or in vivo fate of AuNMs, particularly in the blood, when they encounter the borderline defenders, primary immune cells such as neutrophils and monocytes. The immune response by primary immune cells (neutrophils) has demonstrated the possible biodegradation of carbon nanomaterials (e.g., carbon nanotubes and graphene) mediated by myeloperoxidase (hMPO) catalysis.^[^ ^3^, ^9^ ^]^ In this regard, investigating the potential biodegradation of AuNMs by neutrophils is highly relevant and crucial.

AuNMs exhibit unique physicochemical properties by varying their size, shape, and morphology. More recently, 2D Au nanosheets (AuNS) attracted huge attention due to their unique near‐infrared (NIR)‐light absorption and applications in nanomedicine.^[^ ^10^, ^11^ ^]^ There are a few reported works on the photothermal therapeutic effect of AuNS combined with other materials, such as graphene oxide (GO) and Se‐doping.^[^ ^12^, ^13^ ^]^ However, the extent of the photothermal properties of AuNS alone has not been well explored.

Hereby, we investigated the biodegradation of AuNs by interacting with neutrophil‐secreted human myeloperoxidase (hMPO), using an in vitro model with neutrophil‐like human myeloid leukemia (HL‐60) cells, which are known to secrete MPO upon activation. Both studies confirmed the possible degradation of AuNS, supported by electron microscopy and spectroscopy studies. Further, the near‐infrared (NIR) photothermal properties of AuNS alone were also studied using triple‐negative breast cancer cells. The potential biodegradability of the AuNS could increase their utility for various biomedical fields.

## 2 Results and Discussion

### 2.1 Synthesis and Characterization of Tryptophan‐Reduced Gold Nanosheets

**Figure 1:**
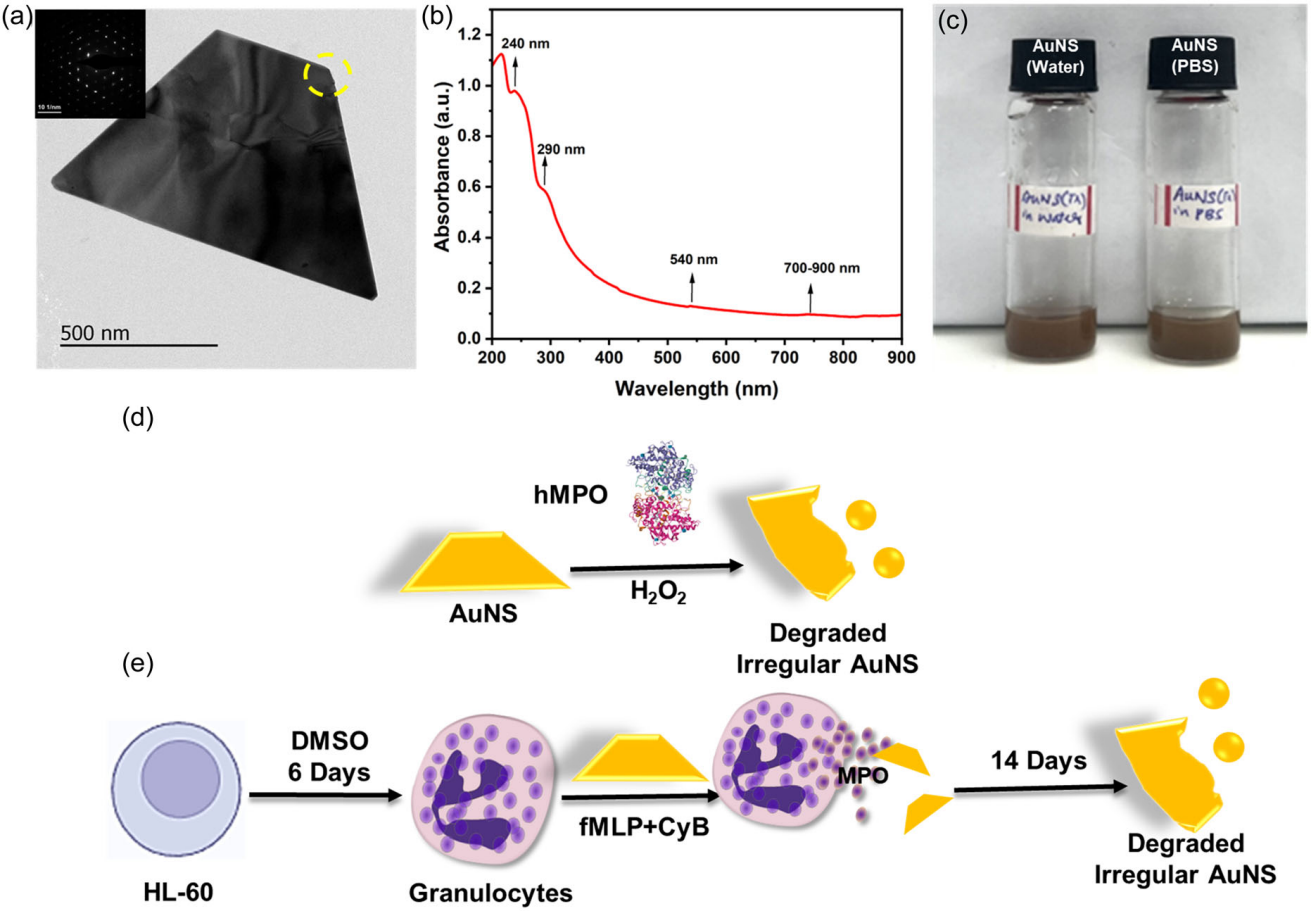
a) Shows an HR‐TEM image of AuNS along with the SAED pattern (inset). b) UV–vis–NIR spectrum of AuNS dispersed in water, c) Digital photos of AuNS dispersed in water and PBS for 2 days, respectively. d) Schematic shows the test‐tube model for biodegradation of AuNS by hMPO, and e) scheme for the in vitro degradation of AuNS using neutrophil‐like HL‐60 cells for 14 days, secreting MPO after activation with N‐formyl‐methionyl‐leucyl‐phenylalanine (*fMLP*) and cytochalasin B (CyB).

First, we synthesized highly water‐dispersible AuNS from HAuCl_4_ and tryptophan (Tr),^[^ ^10^ ^]^ where Tr acts as a reducing agent and surfactant to get a stable aqueous colloidal solution of AuNS. High‐resolution transmission electron microscopy (HR‐TEM) analysis confirmed the flat and sheet‐like morphology of the AuNS in hexagonal or triangular shape with well‐defined edges (**Figure** **1**a), and the selected area diffraction (SAED) pattern confirmed the single crystallinity of AuNS (inset, Figure 1a). In addition to the AuNS, a few spherical nanoparticles (NPs) were also observed (Figure S1, Supporting Information). Furthermore, UV–vis–NIR spectroscopy analysis of AuNS revealed peaks at ≈240 nm, corresponding to the π–π* transitions in the indole ring of tryptophan. Notably, the broad absorption in the NIR region (700–900 nm) confirmed the successful formation of 2D AuNS (Figure 1b).^[^ ^14^ ^]^ Next, the average size of AuNS was determined to be 691.4 ± 168.7 nm using dynamic light scattering (DLS) analysis (Figure S2, Supporting Information). The average lateral size of AuNS, as determined from TEM images, was 696.38 ± 266.7 nm (*n* = 20). Thus, the size obtained from DLS analysis correlates with that of HR‐TEM images. The size appears to be larger than that of the reported sheets (465 ± 130 nm).^[^ ^10^ ^]^ Next, the thickness of the sheets was analyzed using atomic force microscopy (AFM). The average thickness of the AuNS was ≈10 nm, confirming a few layers of AuNS (Figure S3, Supporting Information). Further, X‐ray diffraction (XRD) analysis displayed characteristic peaks (111), (200), (220), (311), and (222) corresponding to the crystallographic planes of face‐centered cubic (fcc) gold (JCPDS 04‐0784), confirming the formation of pure crystalline gold (Figure S4, Supporting Information).^[^ ^15^, ^16^ ^]^ Overall, the XRD data correlate with the SAED pattern (Figure 1a, inset) to confirm the single crystallinity of AuNS. Further, the zeta potential of aqueous AuNS is −36.8 ± 8.18 mV (Figure S5, Supporting Information), indicating moderate colloidal stability. Next, the colloidal stability of AuNS was analyzed by dispersing the sheets in fetal bovine serum (FBS), phosphate‐buffered saline (PBS), and water for 2 days. From the UV–vis–NIR spectroscopic analysis of AuNS in water and PBS, there was only a slight variation in the peak intensities after 2 days. Also, no changes in the intensity of the brown color were observed (Figure 1c and S6, Supporting Information). According to DLS and zeta potential analysis at different time periods (0, 10, 24, and 48 h), a slight increase in size and a reduction in zeta potential value were observed with increasing time (Figure S7 and S8, Supporting Information). This observation also confirms the moderate stability of AuNS over a 48‐h time period in different dispersants.

### 2.2 Test‐Tube Model Biodegradation of AuNS Using Human Myeloperoxidase Enzyme

**Figure 2:**
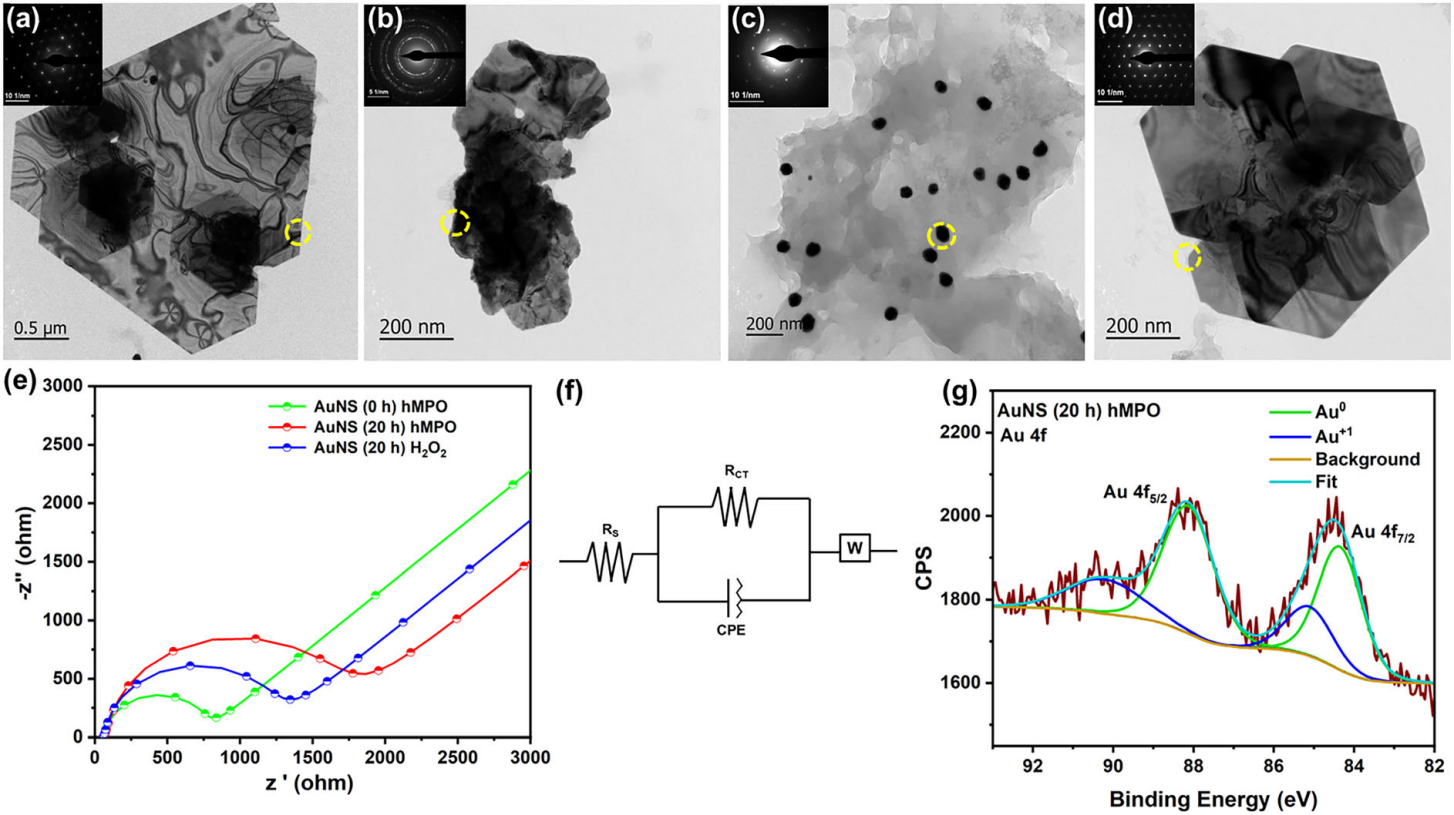
High resolution transmission electron microscopy (HR‐TEM) images a) AuNS (0 h) hMPO, b,c) AuNS (20 h) hMPO (white arrows indicate the degraded parts), and d) AuNS (20 h) H_2_O_2_, and the corresponding SAED patterns are shown in the inset of the respective images. e) Nyquist plots for AuNS (0 h) hMPO, AuNS (20 h) hMPO, and AuNS (20 h) H_2_O_2_ obtained from EIS, and f) the equivalent circuit used to fit the EIS data, where the circuit consists of a parallel combination of resistance and a constant phase element (CPE) connected in series with a Warburg element (*W*, diffusive process), *R*_S_ denotes the uncompensated resistance, and *R*_CT_ corresponds to the resistance to electron transfer imposed by AuNS. g) The XPS data of Au 4*f* XPS spectra of AuNS (20 h) hMPO, respectively.

First, the test‐tube model biodegradation of AuNS was performed by incubating with isolated hMPO in the presence of 200 μM H_2_O_2_ and 140 mM NaCl, where H_2_O_2_ was added once per h up to 20 h (Figure 1d). Next, HR‐TEM was employed to understand the morphological changes of AuNS at different time points (**Figure** **2**a–d). Initially, AuNS were flat with distinct edges in a hexagonal or triangular shape, exhibiting single crystallinity (Figure 2a and S9a,b, Supporting Information) at 0 h (inset, Figure 2a). However, these sheets were found to be significantly different after 20 h of hMPO treatment (Figure 2b and S9c,d, Supporting Information). After analyzing 20 sheets in total, 15 sheets appeared to have undergone morphological changes (550.61 ± 280.85 nm). In addition to the damaged sheets, more spherical NPs were observed around the damaged sheets (Figure 2c), which could be due to the breakage of the AuNS. From an average of 10 images, the number of nanoparticles was 127 with an average size of 64.33 ± 41.22 nm. The distinct morphology, characterized by triangular shapes with sharp edges, was altered to an irregular morphology (Figure 2b), where the edges appear to be damaged (broken). The damage to the edges was also supported by changes observed in the SAED analysis (Figure 2b, inset), which exhibited polycrystallinity, unlike the single crystallinity at 0 h (Figure 2a, inset). Further, such damages to AuNS were not observed for the samples treated only with H_2_O_2_ without hMPO for 20 h (Figure 2d and S9e,f, Supporting Information), including the single crystallinity of the sharp edges (SAED pattern, Figure 2d, inset). After analyzing 20 sheets in total, only six sheets appeared to have undergone morphological changes (692 ± 334.35 nm). Moreover, the average (*n* = 10) of only 28 nanoparticles (Figure S10, Supporting Information) (108.5 ± 30.41 nm) was present in the sample. Such changes in the morphology of nanosheets could be due to the oxidation of AuNS by hMPO, which generates reactive radical intermediates and hypochlorous acid (HOCl).

To further support the transmission electron microscopy (TEM) analysis, electrochemical impedance spectroscopy (EIS) was employed to investigate the possible oxidation of AuNS following hMPO treatment. EIS is used to investigate metal corrosion (oxidation) as electrochemical phenomena occur at the interface of the electrode (coated with the testing material) and the electrolyte.^[^ ^17^ ^]^ The working electrode was drop‐casted with the AuNS (treated with hMPO for 0 or 20 h or only H_2_O_2_), then the electrochemical impedance (Z′, ohm) was measured. Next, the Nyquist plots (negative imaginary impedance, ‐Z″ versus the real part of the impedance, Z′) were analyzed to investigate the electrochemical phenomena (Figure 2e). The observed semicircle at the beginning of the plot relates to the charge transfer process, where the larger the diameter of the semicircle, the higher the resistance of the material (coated on the electrode).^[^ ^17^, ^18^ ^]^ The plot of the hMPO‐treated AuNS had the highest resistance compared to other samples. Furthermore, from the extrapolation of the Nyquist plot by fitting it with the equivalent circuit (Figure 2f), a key parameter, R_CT_ (charge transfer resistance, related to the oxidation of coated materials), was measured. The R_CT_ value is 1444 Ω for the AuNS treated with hMPO for 20 h, significantly higher than that of AuNS treated with hMPO for 0 h (655.9 Ω) and H_2_O_2_‐treated (1090 Ω). The higher R_CT_ value measured for the hMPO‐treated sample confirmed that oxidation/degradation of AuNS occurred. Earlier, EIS was employed to understand the degradation of g‐C_3_N_4_.^[^ ^17^ ^]^ Overall, impedance results strongly support the TEM analysis, confirming the oxidation of AuNS by hMPO. X‐ray photoelectron spectroscopy (XPS) analysis was performed to gain more insights into the oxidation of AuNS (Figure 2g and S11, Supporting Information). The characteristic peaks corresponding to possible spin states of the Au 4*f* electrons after spin‐orbital splitting in the high‐resolution Au 4*f* spectra are Au 4*f*_7/2_ and 4*f*_5/2_, where 4*f*_7/2_ for Au^0^ is between 83.9 and 84.4 eV (binding energy).^[^ ^14^ ^]^ The deconvoluted 4*f* spectrum of hMPO‐treated AuNS shows peaks at 85.11 (Au^I^ 4*f*_7/2_) and 90.97 eV (4*f*_5/2_) (Figure 2g), and peaks present at 84.35 and 88.12 eV correspond to Au^0^.^[^ ^19^ ^]^ The presence of peaks corresponding to both Au^I^ and Au^0^ for the hMPO‐treated AuNS confirmed the possible oxidation of AuNS; such oxidation was observed for AuNPs by ROS in the fibroblast cells incubated for 6 months.^[^ ^8^ ^]^

### 2.3 In Vitro Biodegradation of AuNS with Neutrophil‐like Cells

**Figure 3:**
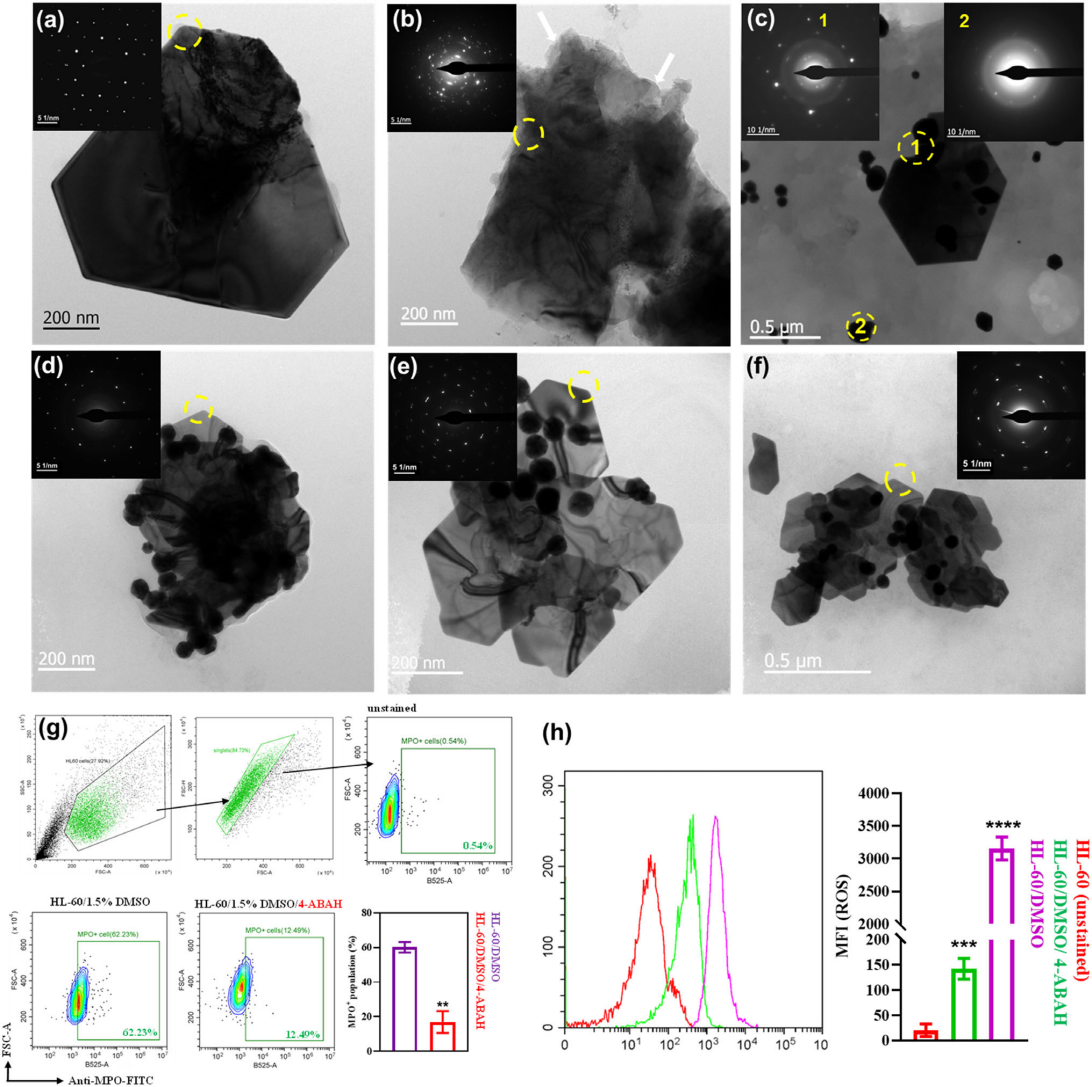
HR‐TEM images of AuNS incubated with HL‐60 cells; a) 0 day, b) 14 days, c) nanosized spherical structure observed in 14‐day sample. 14‐day control, d) AuNS/Cells. e,f) AuNS/Cells/4‐ABAH (100 μM). The degraded parts are indicated with white arrows. The SAED pattern of the circled spots is given in the inset of the respective figures. g) Flow cytometry gating strategy and representative MPO^+^ cell plots for control and 4‐ABAH conditions. h) MPO‐associated ROS activity assessed by DCFDA fluorescence histograms with corresponding quantitative analysis. Data are presented as mean ± SD from at least three independent experiments. Statistical significance was determined by two‐way ANOVA with Tukey's multiple comparisons test (^**^ *P* < 0.01; ^***^ *P* < 0.001; ^****^ *P* < 0.0001).

To support the test‐tube model degradation, the in vitro biodegradability of AuNS was assessed using neutrophil‐like cells that secrete MPO after treatment with fMLP and CyB for 14 days (Figure 1e). First, HL‐60 cells were differentiated into neutrophil‐like cells by the addition of 1.5% dimethyl sulfoxide (DMSO). Robust differentiation of HL‐60 cells into a neutrophil‐mimetic phenotype was first substantiated to authenticate the physiological relevance of the degradation paradigm. Confocal 4′,6‐diamidino‐2‐phenylindole (DAPI) imaging revealed a hallmark nuclear transition from unsegmented nuclear morphology to multilobulated architectures characteristic of terminal granulocytic maturation following exposure to 1.5% DMSO (Figure S12, Supporting Information). Complementary flow cytometric analyzes corroborated this maturation trajectory, evidenced by a pronounced G_0_/G_1_ cell‐cycle arrest, downregulation of CD71, and near‐uniform CD11b expression, collectively aligning with canonical neutrophil differentiation profiles. Critically, Annexin V interrogation revealed negligible apoptotic induction, confirming that the differentiation protocol yielded a viable, functionally competent effector population rather than an artefactual cytotoxic state (Figure S13–S16, Supporting Information). Following activation, the differentiated HL‐60 milieu generated an MPO‐rich oxidative microenvironment. Previously, neutrophil‐like cells were used to investigate the biodegradability of GO.^[^ ^20^ ^]^ Herein, AuNS were incubated with neutrophil‐like cells for 14 days. HR‐TEM analysis of the resulting AuNS (AuNS/Cell/Activators) revealed that nanosheets lost their regular hexagonal or trigonal morphology with sharp edges (**Figure** **3**b, Figure S17a,b, Supporting Information), unlike at 0 h (Figure 3a). Especially, the sharp edges of AuNS were turned into soft and more wrinkled, and some parts of the edges appeared fragmented (broken). Control samples were also performed with AuNS along with activated cells (AuNS/Cells, Figure 3d and S17c,d, Supporting Information), pharmacologic MPO inhibitor 4‐aminobenzoic acid hydrazide (4‐ABAH, (AuNS/Cells/4‐ABAH) and AuNS with Roswell Park Memorial Institute (RPMI) media (AuNS/Media, Figure S17i, Supporting Information). Control experiments with the inhibitor were conducted at 20 μM (Figure S17e,f, Supporting Information) and 100 μM per well (Figure 3e,f and S17g,h, Supporting Information) concentrations of 4‐ABAH. After analyzing 20 sheets, an average of 2 and 3 sheets appeared to be degraded in the AuNS/Cells and AuNS/Cells/4‐ABAH (100 μM) conditions, respectively. Whereas an average of 10 sheets (352.30 ± 86.11 nm) appeared to be degraded or deformed in the AuNS/Cell/Activators case. The average size of the sheets was 417.06 ± 66.08 and 453.1 ± 210.99 nm in AuNS/Cells and AuNS/Cells/4‐ABAH (100 μM). Similarly, 50 and 68 nanosized Au nanoparticles (*n* = 10) with an average size of 120.7 ± 63.25 nm and 90.47 ± 91.54 nm were found in the control samples AuNS/Cells and AuNS/Cells/4‐ABAH (100 μM), respectively. In the case of AuNS/Cells/4‐ABAH (20 μM), 3 sheets appeared to be degraded compared to 20 sheets (547.17 ± 262.62 nm), and a total of 49 nanoparticles, with an average size of 119.13 ± 81.45 nm, were also observed alongside the sheets. At the same time, an average of 113 AuNPs (*n* = 10) were observed in AuNS/Cells/Activators with an average size of 53.16 ± 31.43 nm. No significant changes were observed in the properties and morphology of the sheets and particles at either inhibitory concentration. In conclusion, more spherical NPs with a smaller size were found in AuNS/Cells/Activators (Figure 3c) compared to the control samples (Figure S17c–i, Supporting Information). This is possibly due to oxidation‐mediated degradation of AuNS.

Furthermore, the SAED analysis of the AuNS treated with cells revealed a polycrystalline nature (SAED 1, inset, Figure 3c), unlike the single‐crystalline nature observed at 0 days (Figure 3a). The SAED analysis of the observed spherical NPs revealed more diffuse rings (SAED 2, inset, Figure 3c), indicating a high polycrystalline nature of the resulting NPs. Such NPs with diffused rings were found for the gold salt crystals (aurosome) that resulted from the degradation of gold NPs in the fibroblast cells.^[^ ^8^ ^]^ Further, energy dispersive spectroscopy (EDS) analysis confirmed that the resulting NPs contain gold (Figure S18, Supporting Information). However, such changes in the morphology of the sheets were not significantly observed for the control samples (Figure 3d), confirming the role of neutrophil‐like cells in the degradation of AuNS. Flow cytometric quantification demonstrated a concomitant reduction in MPO^+^ cell burden under inhibitor treatment (Figure 3g–h), while diichlorodihydrofluorescein diacetate (DCFDA) fluorescence profiling revealed a substantial attenuation of ROS output, functionally validating the efficacy of MPO blockade and excluding significant contributory roles for NOX‐derived ROS. This confirms the hMPO‐mediated degradation pathway. All these changes in the morphology and the resulting polycrystallinity of the edges and generated NPs strongly support the possible biodegradation of AuNS by neutrophil‐like cells via MPO catalysis.

### 2.4 Mechanism of Biodegradation of AuNS

**Figure 4:**
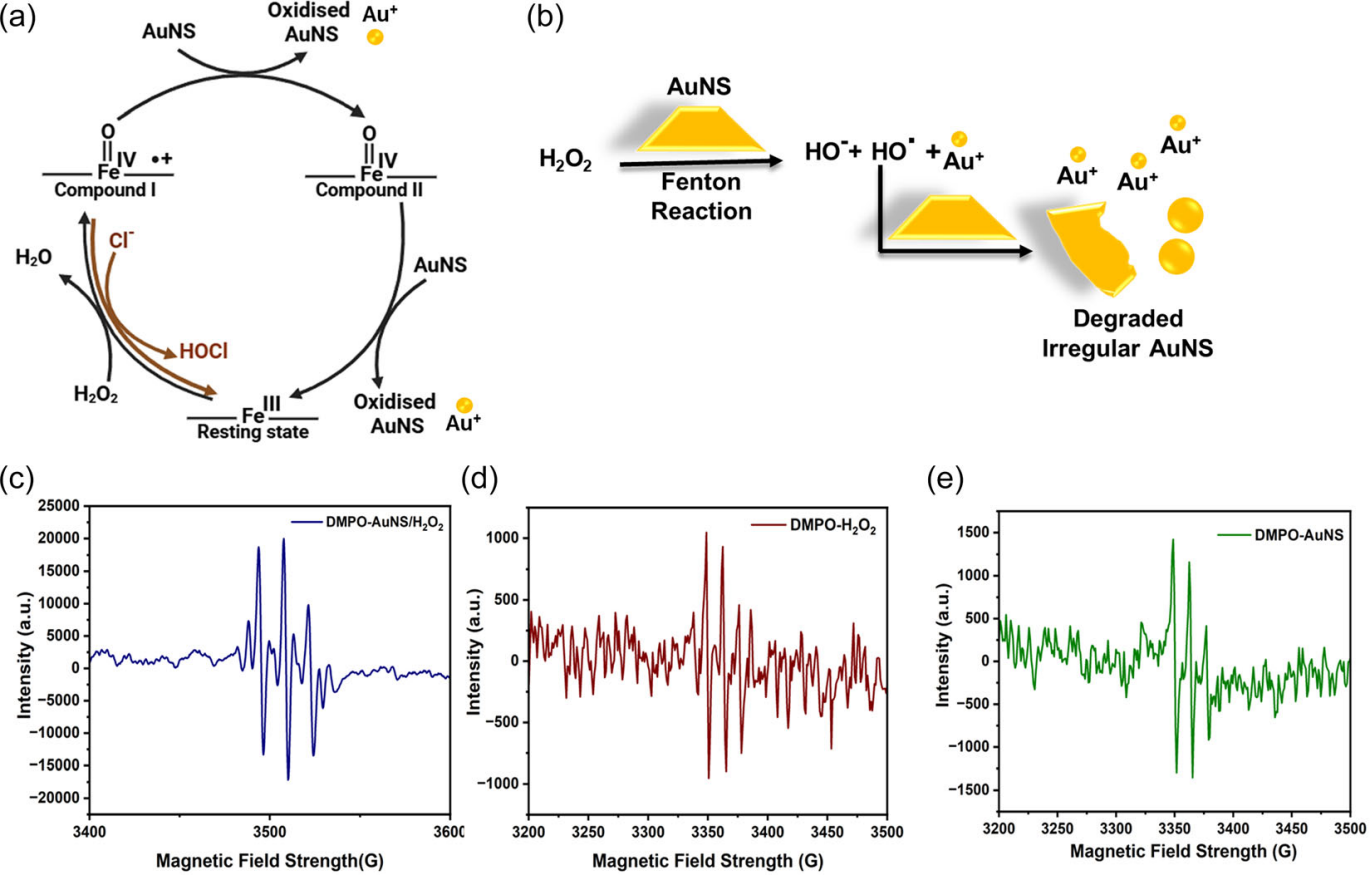
Mechanism of biodegradation of AuNS: a) Catalytic peroxidase cycle of hMPO. In the presence of H_2_O_2_, the enzyme undergoes two‐electron oxidation to form compound I (ferryl oxo iron porphyrin π cation radical) and can return directly to the ferric resting state by converting Cl^‐^ to hypochlorous acid. The AuNS undergoes one electron oxidation in the compound I to compound II (ferryl oxo iron) reduction step and the compound II to resting state reduction step.^[^ ^22^ ^]^ b) Schematic representation of Fenton‐like reaction happening in the presence of H_2_O_2_ by AuNS, where the resulting OH^.^ could degrade AuNS, and c–e) EPR spectrum corresponding to DMP‐OH adduct in AuNS/H_2_O_2_, H_2_O_2_, and AuNS, respectively.

In vitro degradation of AuNS in neutrophil‐like cells is supported by the previous studies of AuNPs biotransformation inside the fibroblasts (6 months), where AuNPs were oxidized to Au^I^ ions by NADPH‐oxidase (NOX) catalysis inside the lysosome. First, NOX catalyzed the generation of superoxide (O_2_^.−^) and converted it to H_2_O_2_ via spontaneous dismutation, then the Fenton reaction by AuNPs mediated the formation of OH^.^ radicals.^[^ ^8^ ^]^ The strong oxidant OH^.^ radicals oxidized the AuNPs into Au^I^, which underwent recrystallization to form a self‐assembled aurosome. The reactive radical intermediates of hMPO and HOCl could be responsible for the oxidation‐mediated degradation of AuNS, since HOCl (plus Cl^−^ ions) is used in the gold leaching.^[^ ^21^ ^]^ The active site of MPO undergoes two‐electron oxidation to form Compound I (Fe^IV^ = O π cation radical) upon reacting with H_2_O_2_, then Compound I is reduced to Compound II (Fe^IV^ = O) and finally to the native state (Fe^III^) (**Figure** **4**a).^[^ ^22^ ^]^ Importantly, Compound I can be reduced back to the native state in the presence of Cl^−^ to form HOCl, which has an even higher oxidation potential (1.48 V) than Compound I and II.^[^ ^23^, ^24^, ^25^ ^]^ Therefore, radical intermediates of hMPO and HOCl could be responsible for the partial degradation of AuNS by forming Au^I^ species (Figure 4a).^[^ ^21^ ^]^ The XPS confirmed the presence of Au^I^ for the hMPO‐treated AuNS (Figure 2g). In addition to hMPO catalysis, OH^.^ radicals could also be generated from H_2_O_2_ via the Fenton‐like reaction mediated by AuNS (Figure 4b), similar to the previous work, where AuNPs mediated the formation of OH^.^ from H_2_O_2_, thereby oxidizing AuNPs.^[^ ^8^ ^]^ Herein, we used electron paramagnetic resonance (EPR) spectroscopy (Figure 4c–e) and the methylene blue (MB) test (Figure S19, Supporting Information) to confirm the generation of hydroxy radicals by AuNS in the presence of H_2_O_2_. First, EPR was performed with the spin trap 5,5‐dimethyl‐1‐pyrrolidone‐N‐oxide (DMPO). The spectrum was recorded for the samples DMPO‐AuNS/H_2_O_2_ to demonstrate the Fenton‐like reaction, as well as in DMPO/H_2_O_2_ and DMPO/AuNS as control samples. The EPR spectrum is composed of a quartet of lines, which is characteristic of the DMPO‐OH adduct.^[^ ^26^ ^]^ These peaks are not very significant in the control samples, which confirms the OH^.^ radical generation in AuNS/H_2_O_2_ due to the Fenton reaction. This observation correlates with the results of the MB test (Figure S19, Supporting Information). Hydroxy radicals have the highest oxidation potential (2.8 V); thus, they could have oxidized to degrade AuNS, such as AuNPs, as reported earlier.^[^ ^8^, ^27^, ^28^ ^]^

### 2.5 Cytocompatibility and NIR Photothermal Therapeutic Effects of AuNS

**Figure 5:**
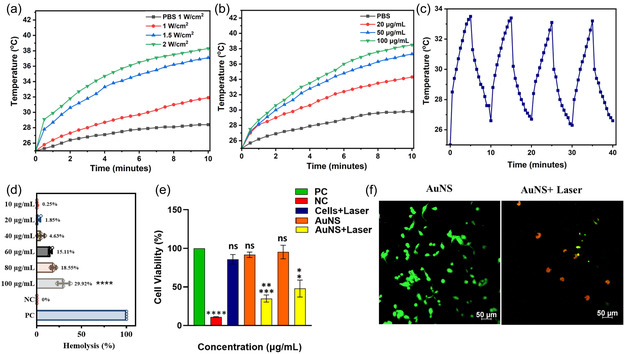
NIR photothermal studies of AuNS with the irradiation of 808 nm NIR laser; a) laser power‐dependent increase in the temperature of AuNS suspension at 50 μg mL^−1^. b) Concentration‐dependent increase in temperature of AuNS with the irradiation of laser at a power density of 1.5 W cm^−2^, and c) photostability of the AuNS in PBS (50 μg mL^−1^) under NIR laser irradiation (1.5 W cm^−2^) for four on/off cycles. d) Hemolysis analysis of AuNS at different concentrations. Hemolytic activity of gold nanosheets (AuNS) was quantified using a standard 1% red blood cell (RBC) lysis assay. The Percentage hemolysis was calculated after background subtraction using NC, i.e., negative control (RBCs + 1X PBS), and normalized to PC, i.e., positive control (RBCs + 1% Triton X‐100), which was set as 100% hemolysis. Data are presented as the mean ± SD from at least four independent experiments (two‐way ANOVA with Tukey's multiple comparisons test, ^****^ *P* < 0.0001). e) In vitro cytotoxicity and photothermal effect of AuNS with and without laser in MDA MB 231 cells, evaluated using MTT assay, each concentration vs positive control (above) and laser irradiated sample concentration vs cells + laser (below). PC = positive control, NC = negative control. Statistical significance was calculated using data from experimental replicates using a two‐tailed unpaired *t*‐test. ^*^ *P* < 0.05, ^**^ *P* < 0.01, ^***^ *P* < 0.001, ^****^ *P* < 0.0001, ns = nonsignificant. Data were represented as mean ± SEM. f) Confocal images of live/dead MDA MB 231 cells incubated with AuNS with and without laser. The green fluorescence indicates the live cells stained with calcein‐AM, and the red fluorescence indicates the dead cells stained with PI. Scale bar: 50 μm.

Further, the NIR photothermal property of AuNS was studied owing to their broad absorption in the NIR region (700–900 nm), which was demonstrated by measuring the rise in temperature upon irradiation with an NIR laser (808 nm) by varying the laser power density (**Figure** **5**a) and the concentration of the AuNS in PBS (Figure 5b). Further, the photothermal stability of AuNS in PBS solution was also examined by irradiating the sample with four “on/off” cycles (5 min each) of laser irradiation at 1.5 W cm^−2^. Even after four cycles, there was not a noticeable change in the photothermal activity of AuNS (Figure 5c), confirming the excellent photothermal stability. We have also studied the photothermal activity of AuNS before and after hMPO/H_2_O_2_ degradation (Figure S20, Supporting Information). The photothermal activity of AuNS appears to be declining after degradation.

Preliminary biocompatibility assessments of gold nanosheets indicated minimal hemolytic liability across escalating AuNS concentrations, underscoring a favorable hemocompatibility profile and supporting nascent claims of biomedical suitability (Figure 5d). Furthermore, the in vitro photothermal effects of AuNS were studied using MDA‐MB‐231 triple‐negative breast cancer cells by irradiating them with an NIR laser. The cell viability of AuNS‐treated cells was decreased below 40% after NIR‐laser irradiation (Figure 5e), compared to control cells, confirming the photothermal killing effect. These results were further supported by Live/Dead imaging (Figure 5f), which demonstrated that most cells were dead after NIR‐laser treatment compared to the control cells. The cytotoxicity studies of AuNS were also examined in the noncancerous HEK 293 T cell line. No significant toxicity was observed for AuNS in HEK 293 T cells with and without NIR irradiation (Figure S21, Supporting Information).

## 3 Conclusions

In conclusion, we successfully demonstrated the possible biodegradation of AuNS using both the test tube and in vitro neutrophil‐like cells mediated by hMPO catalysis. HR‐TEM, impedance and XPS studies confirmed oxidation‐mediated degradation of AuNS to Au^I^ species, where reactive hMPO intermediates, HOCl and hydroxy radicals generated by Fenton reaction could be responsible for the degradation of Au nanosheets. Also, AuNS was successfully utilized for NIR photothermal therapy to kill cancer cells. Biodegradation results could be crucial to understanding the long‐term fate of gold nanomaterials in the body, especially in the blood. Overall, results from the test tube and in vitro models clearly demonstrated the partial biodegradation of AuNS by hMPO. MPO is present mainly in the neutrophils (blood) and in small amounts in the macrophages (tissues). Further studies should be conducted regarding their application and degradation mechanisms, both in vitro and in vivo.

## 4 Experimental Section

4.1

4.1.1

##### Materials

Gold(III) chloride trihydrate (HAuCl_4_.3H_2_O), hMPO derived from human neutrophil, and hydrogen peroxide (H_2_O_2_, 30% aqueous solution), diethylenetriamine pentaacetic acid (DTPA), NaCl, NaH_2_PO_4_.2H_2_O, Na_2_H_2_PO_4_.2H_2_O, DMSO, and pharmacological MPO inhibitor 4‐aminobenzoicacid hydrazide (4‐ABAH) were purchased from Sigma‐Aldrich. L‐Tryptophan was purchased from Tokyo Chemicals (TCI). The Milli‐Q water was used in all the experiments with resistivity higher than 18 MΩ. 3‐(4,5‐dimethylthiazol‐2‐yl)‐2,5‐diphenyltetrazolium bromide (MTT) dye, calcein‐AM, propidium iodide (PI), N‐formyl‐methionyl‐leucylphenylalanine (*fMLP*) and cytochalasin B (CyB) were purchased from Invitrogen. HEK 293 T cells and MDA MB 231 cells were gifted from Prof. Reji Varghese's lab, School of Chemistry, IISER TVM. HL‐60 cells were obtained from the National Centre for Cell Science (NCCS), Pune. The Human MPO antibody was purchased from ELK Biotechnology. H2DCFDA (for ROS detection) and Annexin‐mCherry kit (for apoptosis) were purchased from Thermo Fischer Scientific. For HL‐60 differentiation, FACS (fluorescence‐activated cell sorting) markers‐Hu CD71 BV786 and Hu CD11B FITC were purchased from BD Biosciences. RPMI 1640 and DMEM media were purchased from Gibco.

##### Synthesis of Tryptophan‐Reduced Gold Nanosheets (AuNS)

All the glassware required for the experiment was cleaned thoroughly with aqua regia and oven‐dried. The AuNS sheets were prepared according to the previous report.^[^ ^10^ ^]^ Briefly, 10 mL of 10 mM HAuCl4·3H_2_O and 10 mL of 5 mM tryptophan were mixed in a 30 mL vial and allowed to stir continuously for 1 h at room temperature, then the resulting solution was left for 72 h at room temperature and lyophilized.^[^ ^10^ ^]^ The synthesized AuNS was characterized using UV–vis–NIR spectroscopy, HR‐TEM, AFM and DLS measurements, including zeta potential.


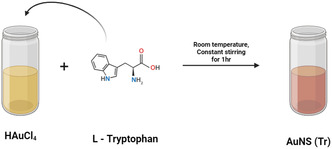


##### HR‐TEM Analysis

For HR‐TEM imaging of AuNS, the samples were drop‐cast on a 300‐mesh carbon‐coated copper grid (Ted Pella, Inc.) and dried for 15–20 min under an infrared (IR) lamp. The samples were imaged using FEI Tecnai G2 F30 S‐Twin TEM 300 kV. The chemical composition of the samples was analyzed using EDS coupled with HR‐TEM. An average of 20 sheets was analyzed using ImageJ software.

##### UV–vis–NIR Spectroscopy

The absorption spectra were recorded using a Shimadzu UV‐3600 vis‐NIR spectrophotometer. The samples were taken in a quartz cuvette of 10 mm path length.

##### AFM Measurements

The gold nanosheet samples were drop‐cast on silicon wafers, dried in a 60 °C oven, and analyzed by JPK Nanowizard 4AFM. The average height and roughness of the sheets were obtained using JPK software.

##### DLS and Zeta Potential Analysis

For DLS and zeta potential analysis of the AuNS, the samples were diluted with deionized (DI) water to create a well‐dispersed suspension before taking the readings. The average of three independent readings was used for each measurement. The analysis was recorded using Zetasizer Nano ZS with a 655 nm laser.

##### Powder X‐Ray Diffraction (PXRD)

PXRD analyses were performed using Panalytical powder XRD (CuKα, *λ* = 1.5406 Å, 40 kV, 40 mA) in a 2*θ* range of 10 to 90° and with a scan rate of 1.8° per min. The AuNS samples were submitted in powder form for analysis.

##### Colloidal Stability of AuNS in PBS Buffer

The colloidal stability of AuNS in PBS buffer was investigated by dispersing 0.2 mg of the lyophilized AuNS in 1 mL of water, 1 mL of 50 × 10^−3^ M PBS, and FBS, and comparing the results after 2 days. The stability was evaluated using UV–vis–NIR absorption spectroscopy, DLS, and zeta potential measurements.

##### Enzymatic Degradation of AuNS by Human Myeloperoxidase (hMPO)

The biodegradation of the as‐synthesized AuNS using the enzyme hMPO was conducted similarly to our previous work.^[^ ^29^ ^]^ Briefly, 0.2 mg of the lyophilized AuNS and 100 μg of hMPO were suspended in 1 mL of 50 mM phosphate buffer containing 140 mM NaCl and 100 μM DTPA, where H_2_O_2_ was added at 200 μM (final concentration) per hour for 5 h. hMPO was renewed every 5 h (5 additions: at 0, 5, 10, 15, and 20 h). The reaction mixture was maintained at 37 °C for 20 h. The 0 h samples were also collected at 0 h. A control experiment was also performed to assess the degradation of AuNS samples by adding H_2_O_2_, using the same protocol. The samples were further analyzed using HR‐TEM, EIS, and XPS.

##### HR‐TEM Analysis

For HR‐TEM analysis, the degraded samples and the control samples were drop‐casted on a 300‐mesh carbon‐coated copper grid and dried for 15 min under an IR lamp. The grids were washed with water for 20 min to remove the salts and again dried. A total of 10 images were analyzed using ImageJ software.

##### EIS

EIS was conducted utilizing an Autolab MSTAT204 potentiostat/galvanostat (Metrohm) with the sample immobilized on glassy carbon electrodes (GCEs). For EIS, a three‐electrode configuration was employed, comprising the Pt wire as the counter electrode (CE), GCE as the working electrode (WE) and Ag/AgCl as the reference electrode (RE), similar to the degradation of g‐C_3_N_4_ sheets.^[^ ^17^ ^]^ AuNS suspensions of degraded and control samples were drop‐casted onto the GCE (electrode area 0.07 cm^2^), and measurements were conducted in a 5 mM potassium hexacyanoferrate (III) solution with a 100 mM PBS electrolyte (containing 100 mM KCl, pH 7.4). A potential of 0.21 V (vs. reference) was applied with a perturbation of 10 mV over a frequency range of 100 kHz to 10 mHz.^[^ ^17^ ^]^ The data analysis and fitting were performed using EC Lab software.

##### XPS

XPS analyses of the samples were performed using Omicron Nanotech XPS in CAE analyzer mode with a 1253.6 eV MgKα excitation source with a pass energy of 50 eV. The degraded samples were drop‐casted on a silicon wafer and dried under an IR lamp for 15 min.

##### In Vitro Degradation of AuNS

The in vitro degradation of AuNS was performed using HL‐60 cells, which were differentiated into neutrophil‐like cells by treating them with 1.5% DMSO for 6 days.^[^ ^20^ ^]^ After differentiation, the cells were seeded into a six‐well plate with a density of 2 × 10^6^ cells well^−1^ and a volume of 2 mL in RPMI 1640 media without phenol red. After seeding, a 30 μg mL^−1^ concentration of AuNS was added into the wells along with activators N‐formyl‐methionyl‐leucylphenylalanine (*fMLP*, 100 nM) and cytochalasin B (*CyB*, 5 μg mL^−1^) and incubated for 14 days. Control wells were also maintained with cells along with *fMLP* and *CyB*, AuNS alone with RPMI 1640 media (AuNS/Media), AuNS with activated cells (AuNS/Cells), and AuNS with activated cells and MPO inhibitor 4‐aminobenzoic acid hydrazide (AuNS/Cells/Inhibitor). The inhibitor was added at a concentration of 20 μM and 100 μM per well.^[^ ^24^ ^]^ The concentration of all components was kept the same for the control samples as well. After incubating for 14 days, all the samples were centrifuged at 1300 rpm for 1 min. The supernatants were collected and characterized using HR‐TEM. The 0th day sample was also collected by preparing AuNS at a concentration of 30 μg mL^−1^ in the media and centrifuging under the same conditions.^[^ ^20^ ^]^

##### HR‐TEM Analysis

For HR‐TEM analysis, the degraded samples and the control samples were drop‐casted on a 300‐mesh carbon‐coated copper grid and dried under an IR lamp for 15 min. The samples were further plasma cleaned to remove any cell debris present. A total of 20 images were analyzed using ImageJ software.

##### EPR Spectroscopy

DMPO (100 mM) was mixed with AuNS dispersion in PBS buffer (0.2 mg mL^−1^). 200 μM concentration of H_2_O_2_ was added, and 50 μL of the mixture was immediately transferred to a capillary tube, and the spectrum was recorded by inserting the capillary tube into the EPR tube. Control samples were also performed with DMPO, DMPO‐H_2_O_2_, and DMPO‐AuNS. The EPR measurements were performed using a Bruker X‐band EPR spectrometer, operating at ≈9.45 GHz at room temperature.

##### MB Test

The hydroxyl radical generation ability of AuNS after treating with H_2_O_2_ (Fenton‐like reaction) was qualitatively examined by the MB test. Briefly, filter paper strips were dipped in an aqueous solution of MB for 10 min and dried. After that, 20 μL of AuNS solution in PBS (AuNS) and in H_2_O_2_ (AuNS/H_2_O_2_) were drop‐casted on the strips and dried. Fenton mixture (FeSO_4_.7H_2_O + H_2_O_2_) and H_2_O_2_ alone were drop‐casted as a control. The color change of the strips was observed and photographed using the digital camera.

##### Flow Cytometry

Flow cytometry analyses were performed on HL‐60 cells following standard gating and acquisition procedures. Cells were collected, washed in PBS, and resuspended in buffer (PBS with 2% FBS). Debris was excluded using FSC‐A versus SSC‐A gating, and singlets were isolated through FSC‐A versus FSC‐H discrimination. Unstained and single‐stained controls were included to establish compensation and define positive gates. For cell‐cycle analysis, cells were fixed in 70% ethanol, treated with RNase A, and stained with PI before acquiring DNA histograms. Apoptosis was assessed by Annexin V staining and quantified on Annexin V versus SSC plots using the same gating hierarchy. For CD71 and CD11b surface marker assessment, cells were incubated with fluorophore‐conjugated antibodies, washed, and analyzed under identical voltage and gating settings. All samples were acquired using consistent voltages and thresholds on Beckman Coulter's Cytoflex LX flow cytometer, and results were processed in CytExpert v2.6, with data presented as mean ± SD from at least three independent experiments.

##### NIR Photothermal Properties of AuNS

NIR photothermal studies of AuNS samples were conducted using an 808 nm NIR‐laser (CNI, part number MDL‐III‐808). Briefly, a 50 μg mL^−1^ AuNS suspension in a quartz cuvette was irradiated with the laser for 10 min at different laser power densities, namely 1, 1.5, and 2 W cm^−^^2^. This is followed by irradiation of different concentrations of AuNS solutions in PBS (20, 50, and 100 μg mL^−1^) with a laser of power density 1.5 W cm^−^^2^. The temperature rise in both cases was noted with a thermocouple probe digital thermometer every 30 s. PBS was taken as a control for both experiments.

To assess the photothermal stability of AuNS, the AuNS solution in PBS (50 μg mL^−1^) was exposed to a laser beam (808 nm, 1.5 W cm^−^^2^) for 5 min. After the laser was switched off and the solution had cooled to ambient temperature for 5 more min, three further cycles of laser irradiation were carried out, one “on” and one “off”. The solution's temperature was measured every 30 s.

Next, to understand the photothermal activity of AuNS before and after hMPO/H_2_O_2_ degradation, the 0 and 20 h samples were irradiated with the NIR laser (1.5 W cm^−2^, 10 min). The temperature rise was measured every 30 s.

##### RBC Hemolysis Assay

Hemolytic activity of gold nanosheets (AuNS) was quantified using a standardized red blood cell lysis assay. Fresh human whole blood was collected inethylenediaminetetraacetic acid (EDTA) tubes and centrifuged at 500X g for 5 min to pellet erythrocytes. The plasma and buffy coat were removed, and the erythrocytes were washed three times with PBS before being diluted to the working concentration (1%). AuNS stock solutions were prepared at 20× the desired final assay concentration. For each test condition, 10 μL of AuNS stock was added to 190 μL of diluted erythrocytes in U‐bottom 96‐well plates, yielding final AuNS concentrations of 10, 20, 40, 60, 80, and 100 μg mL^−1^. RBCs + PBS served as the negative control (NC), and RBCs + 0.1% Triton X‐100 were used as the positive control (PC) for complete hemolysis. Plates were incubated at 37 °C for 1 h, followed by centrifugation at 500X g for 5 min to pellet intact RBCs. 100 μL of supernatant from each well was transferred to a clear flat‐bottom plate, and absorbance was measured at 540 nm using a plate reader. Background absorbance from the negative control was subtracted from all readings. The percentage hemolysis was calculated relative to the positive control, which was defined as 100% lysis. AuNS‐induced hemolysis at all concentrations was quantified using this normalized absorbance method and expressed as mean ± SD from biological quadruplets.

##### In Vitro Cytotoxicity and NIR Photothermal Effect

An MTT assay was used to examine the cytotoxicity of AuNS with the triple‐negative breast cancer cell line MDA MB‐231, both with and without NIR‐laser irradiation. In a 96‐well plate, cells were seeded at a density of 5 × 10^4^ cells per well in dulbecco's modified eagle medium (DMEM) with 10% FBS. The cells were incubated for 24 h at 37 °C with 5% CO_2_. Different concentrations of AuNS (50 μg mL^−1^ and 100 μg mL^−1^) were diluted in DMEM and added to various wells. Positive control (untreated cells) and negative control (Triton X‐100) were also maintained. After 5 h of incubation with AuNS at concentrations of 50 and 100 μg mL^−1^, the cells were irradiated twice with the 808 nm NIR‐laser for 10 min with a time gap of 20 min at a power density of 1.5 W cm^−2^ and further incubated for 24 h. After 24 h of incubation, cells were rinsed with PBS solution, and then 100 μL of a freshly made 0.5 mg mL^−1^ MTT was added. Triplicate was performed. The culture media were discarded after 4 h, and 50 μL of DMSO was added to each well to dissolve the formazan crystal. The samples were shaken for 15 min, and the absorbance was measured at 570 nm using a microplate reader. The same experimental conditions were maintained to conduct the MTT analysis of AuNS in non‐cancerous HEK 293 T cells. The absorbance was correlated with cell viability, assuming 100% viability for untreated control cells (positive control). The absorption spectrum for MTT analysis is recorded using a BioTek Synergy H1 microplate reader.

##### Live/Dead Imaging

MDA MB 231 (1.0 × 10^5^) cells were seeded into a 24‐well culture dish plate with a glass cover slip in it. After incubating the cells for 24 h, the culture medium was discarded. Then the cells were treated with AuNS (50 μg mL^−1^). After 5 h of incubation, one set of the treated cells was irradiated by an 808 nm NIR laser (1.5 W cm^−2^, 10 min) repeatedly with a gap of 20 min, and another set with AuNS was kept as a control. After 10 h of incubation, the cells were gently washed with PBS, and calcein‐AM and PI were added at a concentration of 10 μg mL^−1^ in PBS. Calecin‐AM will stain the live cells green, and PI will stain the dead cells red. The samples were kept in the dark for 0.5 h, and then the dye was removed, and one more PBS wash was given. Finally, the sample images were measured by a confocal laser scanning microscope (CLSM, Nikon).

## Supporting Information

Supporting Information is available from the Wiley Online Library or from the author.

## Conflict of Interest

The authors declare no conflict of interest.

## Supporting information

Supplementary Material

## Data Availability

The data that support the findings of this study are available in the supplementary material of this article.

## References

[1] X. Yang , M. Yang , B. Pang , M. Vara , Y. Xia , Chem. Rev. 2015, 115, 10410. 26293344 10.1021/acs.chemrev.5b00193

[2] A. Balfourier , J. Kolosnjaj‐Tabi , N. Luciani , F. Carn , F. Gazeau , Proc. Natl. Acad. Sci. 2020, 117, 22639. 32900936 10.1073/pnas.2007285117 PMC7502769

[3] S. Vranic , R. Kurapati , K. Kostarelos , A. Bianco , Nat. Rev. Chem. 2025. 10.1038/s41570-024-00680-5 39794485

[4] A. Balfourier , A.‐P. Marty , F. Gazeau , ACS Nanosci. Au 2023, 3, 46. 36820094 10.1021/acsnanoscienceau.2c00035 PMC9936776

[5] C. M. Goodman , C. D. McCusker , T. Yilmaz , V. M. Rotello , Bioconjugate Chem. 2004, 15, 897. 10.1021/bc049951i 15264879

[6] Y. Pan , S. Neuss , A. Leifert , M. Fischler , F. Wen , U. Simon , G. Schmid , W. Brandau , W. Jahnen‐Dechent , Small 2007, 3, 1941. 17963284 10.1002/smll.200700378

[7] W. G. Kreyling , A. M. Abdelmonem , Z. Ali , F. Alves , M. Geiser , N. Haberl , R. Hartmann , S. Hirn , D. J. de Aberasturi , K. Kantner , G. Khadem‐Saba , J.‐M. Montenegro , J. Rejman , T. Rojo , I. R. de Larramendi , R. Ufartes , A. Wenk , W. J. Parak , Nat. Nanotechnol. 2015, 10, 619. 26076469 10.1038/nnano.2015.111

[8] A. Balfourier , N. Luciani , G. Wang , G. Lelong , O. Ersen , A. Khelfa , D. Alloyeau , F. Gazeau , F. Carn , Proc. Natl. Acad. Sci. 2020, 117, 103. 31852822 10.1073/pnas.1911734116 PMC6955300

[9] M. Cao , R. Cai , L. Zhao , M. Guo , L. Wang , Y. Wang , L. Zhang , X. Wang , H. Yao , C. Xie , Y. Cong , Y. Guan , X. Tao , Y. Wang , S. Xu , Y. Liu , Y. Zhao , C. Chen , Nat. Nanotechnol. 2021, 16, 708. 33603238 10.1038/s41565-021-00856-w

[10] A. Baral , F. Cavalieri , S. Chattopadhyay , M. Ashokkumar , ACS Sustainable Chem. Eng. 2021, 9, 13953.

[11] Q. Cai , C. Wang , S. Gai , P. Yang , ACS Appl. Mater. Interfaces 2022, 14, 3809. 35015499 10.1021/acsami.1c21307

[12] S. He , J. Li , M. Chen , L. Deng , Y. Yang , Z. Zeng , W. Xiong , X. Wu , Int. J. Nanomed. 2020, 15, 8451. 10.2147/IJN.S265134 PMC7605662 33149586

[13] Y. Kong , Q. He , H. Zhang , H. Sun , Y. Wang , X. Wu , Y. Ma , Y. Zheng , J. Mater. Chem. C 2024, 12, 19515.

[14] Z. Zhang , D. Ni , F. Wang , X. Yin , S. Goel , L. N. German , Y. Wang , J. Li , W. Cai , X. Wang , Nano Res. 2020, 13, 3217. 34295454 10.1007/s12274-020-2990-7 PMC8291290

[15] Z. Li , Z. Liu , J. Zhang , B. Han , J. Du , Y. Gao , T. Jiang , J. Phys. Chem. B 2005, 109, 14445. 16852818 10.1021/jp0520998

[16] M. Zhou , M. Lin , L. Chen , Y. Wang , X. Guo , L. Peng , X. Guo , W. Ding , Chem. Commun. 2015, 51, 5116. 10.1039/c4cc10040a 25714372

[17] L. Didonè , Y. Shin , A. Silvestri , M. Prato , S. Park , A. Bianco , Nanoscale 2024, 16, 1304. 38131206 10.1039/d3nr04502a

[18] F. Sun , X. Peng , X. Bai , Z. Chen , R. Xie , B. He , P. Han , RSC Adv. 2022, 12, 16979. 35755583 10.1039/d2ra01634f PMC9172561

[19] A. Y. Klyushin , T. C. R. Rocha , M. Hävecker , A. Knop‐Gericke , R. Schlögl , PCCP 2014, 16, 7881. 24643747 10.1039/c4cp00308j

[20] X. He , D. L. White , A. A. Kapralov , V. E. Kagan , A. Star , Anal. Chem. 2020, 92, 12880. 32803946 10.1021/acs.analchem.0c01380

[21] M. Baghalha , Int. J. Miner. Process. 2007, 82, 178.

[22] G. P. Kotchey , S. A. Hasan , A. A. Kapralov , S. H. Ha , K. Kim , A. A. Shvedova , V. E. Kagan , A. Star , Acc. Chem. Res. 2012, 45, 1770. 22824066 10.1021/ar300106h PMC3473158

[23] V. Kagan , N. Konduru , W. Feng , B. Allen , J. Conroy , Y. Volkov , I. Vlasova , N. Belikova , N. Yanamala , A. Kapralov , Y. Tyurina , J. Shi , E. Kisin , A. Murray , J. Franks , D. Stolz , P. Gou , J. Klein‐Seetharaman , B. Fadeel , A. Star , A. Shvedova , Nat. Nanotechnol. 2010, 5, 354. 20364135 10.1038/nnano.2010.44 PMC6714564

[24] R. Kurapati , C. Backes , C. Ménard‐Moyon , J. N. Coleman , A. Bianco , Angew. Chem. Int. Ed. 2016, 55, 5506. 10.1002/anie.201601238 27010606

[25] R. Kurapati , S. P. Mukherjee , C. Martín , G. Bepete , E. Vázquez , A. Pénicaud , B. Fadeel , A. Bianco , Angew. Chem. Int. Ed. 2018, 57, 11722. 10.1002/anie.201806906 30006967

[26] S. K. Han , T.‐M. Hwang , Y. Yoon , J.‐W. Kang , Chemosphere 2011, 84, 1095. 21561642 10.1016/j.chemosphere.2011.04.051

[27] D. Elgrabli , W. Dachraoui , H. d. Marmier , C. Ménard‐Moyon , D. Bégin , S. Bégin‐Colin , A. Bianco , D. Alloyeau , F. Gazeau , Sci. Rep. 2017, 7, 40997. 28120861 10.1038/srep40997 PMC5264386

[28] K. Swetha , S. Sudeshna , F. A. L. S. Silva , F. C. Silva , B. Freitas , J. A. C. Incorvia , J. R. Fernandes , A. Jayaraj , S. Banerjee , N. Sadananda Singh , F. D. Magalhães , A. M. Pinto , R. Kurapati , Carbon 2024, 229, 119486.

[29] R. Kurapati , J. Russier , M. A. Squillaci , E. Treossi , C. Ménard‐Moyon , A. E. Del Rio‐Castillo , E. Vazquez , P. Samorì , V. Palermo , A. J. S. Bianco , Small 2015, 11, 3985. 25959808 10.1002/smll.201500038

