# Supplementary material for: Biodegradable Aurum: Gold Nanosheets Undergo Biodegradation by Neutrophil Myeloperoxidase
Source: Small Sci. 2026 Apr 24;6(4):e202500491. doi: 10.1002/smsc.202500491 (PMC13116356; doi:10.1002/smsc.202500491)
Supplement: Supplementary file 1 — Supplementary Material [file SMSC-6-e202500491-s001.pdf]

Bio-Degradable Aurum: Gold Nanosheets Undergo Biodegradation by Neutrophil Myeloperoxidase

Pavithra Kurungottu[a], Aravind Kannoth Anilkumar[a], Soumyadeep Poddar[b], K Swetha[a], Parvathy Anil[a], Srinivasa Reddy Bonam[b,c], Rajendra Kurapati[a]**

Dedicated to Prof. Alberto Bianco, for completing 25 years at the CNRS-IBMC, Strasbourg, France

[a]School of Chemistry, Indian Institute of Science Education and Research, Maruthamala PO, Vithura, Thiruvananthapuram, 695551, India

[b]Vaccine Immunology Laboratory, Department of Applied Biology, CSIR-Indian Institute of Chemical Technology, Hyderabad 500007, India

[c]Academy of Scientific and Innovative Research, Ghaziabad 201002, India

*E-mail: bonamsr.iict@csir.res.in; rkurapati@iisertvm.ac.in;

Dedicated to Prof. Alberto Bianco, for completing 25 years at the CNRS-IBMC, Strasbourg, France

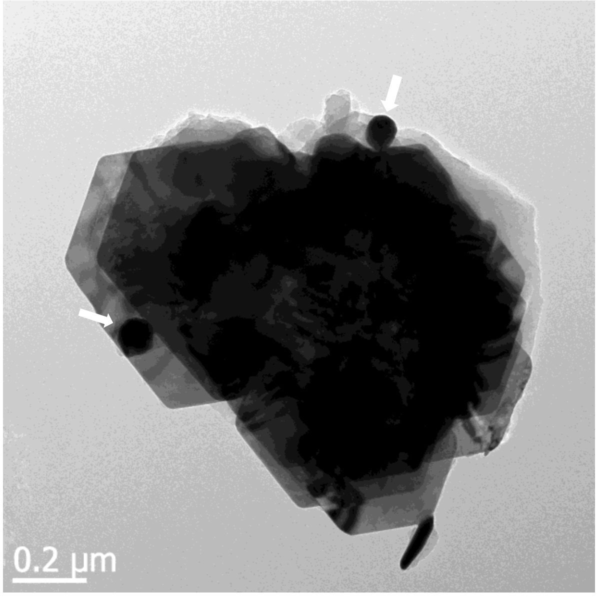

Figure S1. The spherical Au nanostructures obtained along with the AuNS are marked with the arrows.

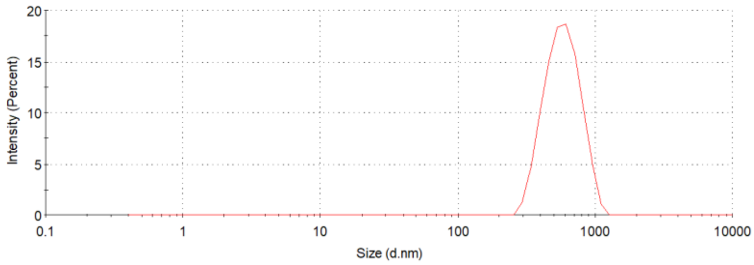

Figure S2. Dynamic light scattering analysis of AuNS in water with an average size of 691.4 ± 168.7 nm in water.

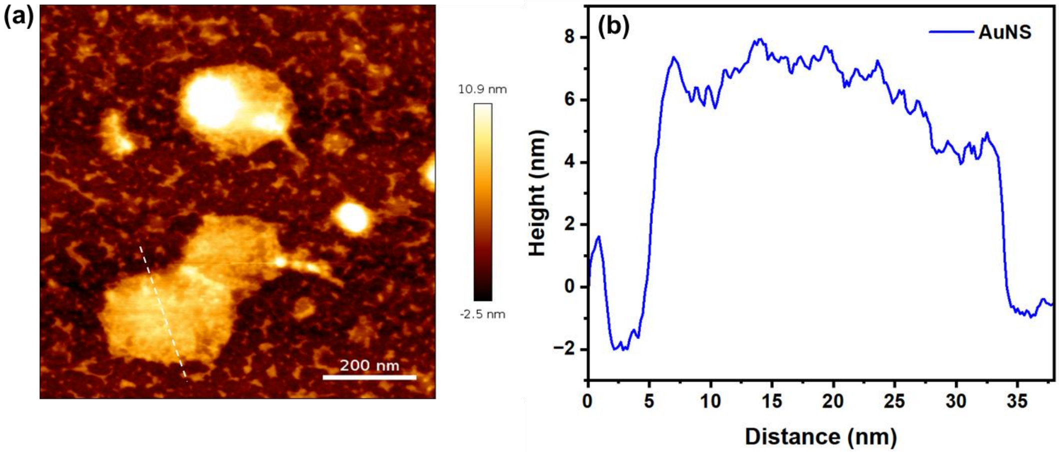

Figure S3. (a) AFM microscope image of AuNS and its corresponding line profile (thickness profile) shown in (b).

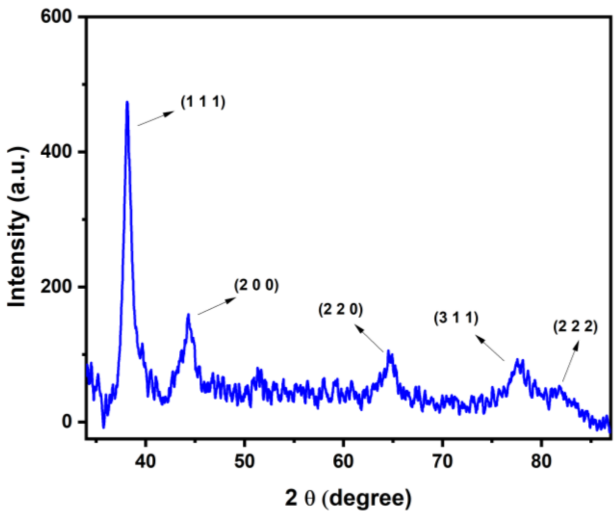

Figure S4. The PXRD pattern of AuNS showed characteristic peaks corresponding to crystalline gold.

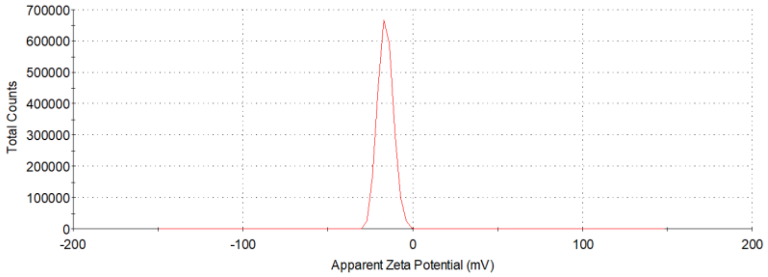

Figure S5. Zetapotential analysis of AuNS in water with the value -36.8 ± 8.18 mV.

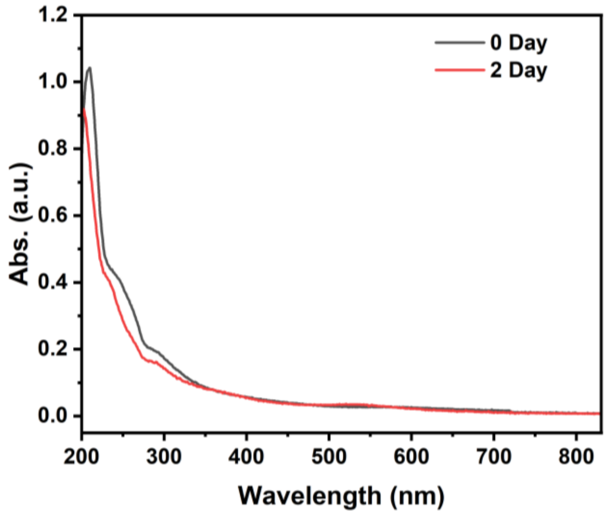

Figure S6. UV-vis-NIR spectra of AuNS dispersed in PBS for 0 and 2 days, respectively.

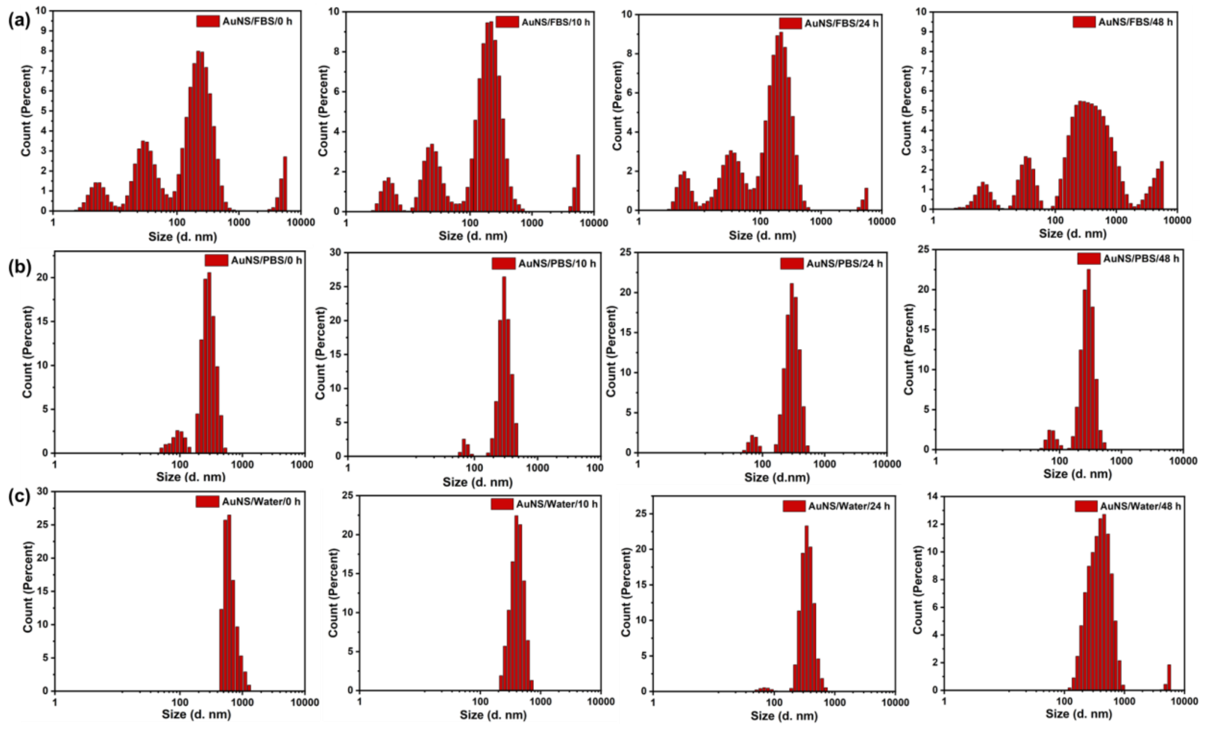

Figure S7: DLS analysis of AuNS in (a) FBS, (b) PBS and (c) water at time intervals of 0, 10, 24 and 48 h, respectively.

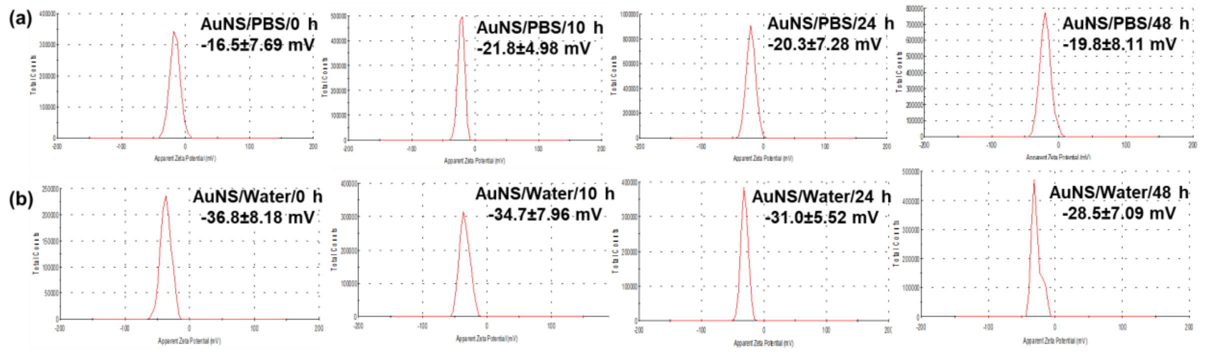

Figure S8: Zetapotential analysis of AuNS in (a) FBS, (b) PBS and (c) water at time intervals of 0, 10, 24, and 48 h, respectively. The zeta potential values of AuNS dispersed in FBS were -12.56 ± 0.2, -11.8 ± 0.1, -8.6 ± 2.77, and -4.33 ± 3.47 mV at 0, 10, 24 and 48 h, respectively.

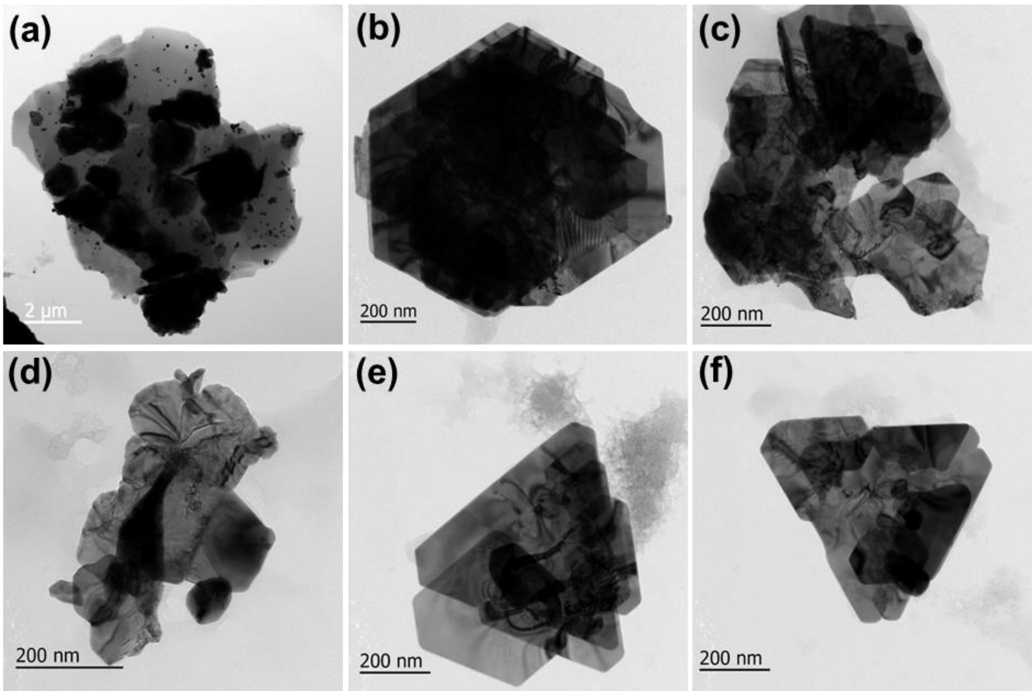

Figure S9. HR-TEM images of (a-b) AuNS(0 h) hMPO, (c-d) AuNS (20 h) hMPO, and (e-f) AuNS (20 h) H_2O_2, respectively.

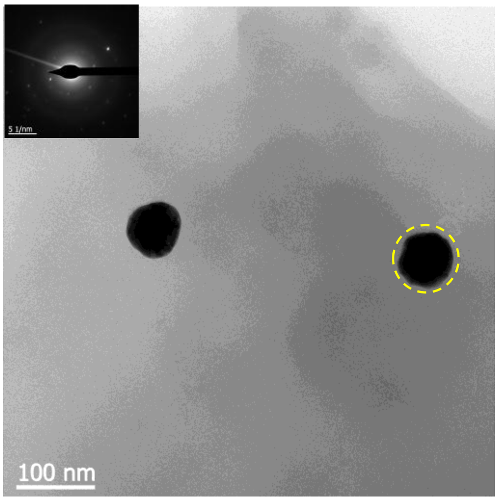

Figure S10. HR-TEM image of AuNS (20 h) H_2O_2 with the SAED pattern in the inset.

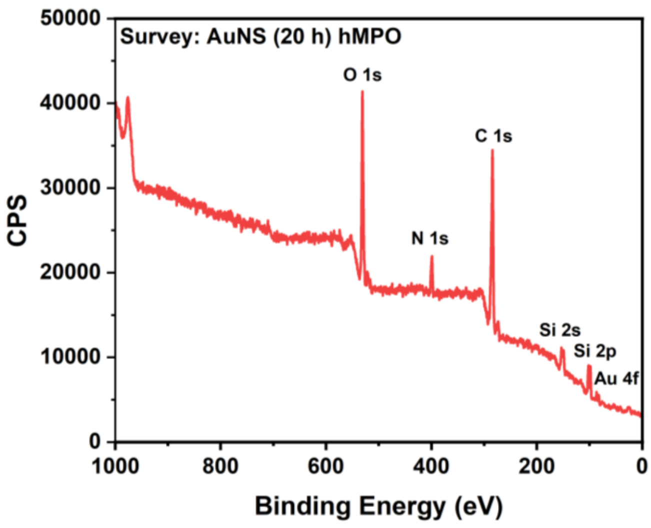

Figure S11. XPS survey spectrum of Au (20 h) hMPO.

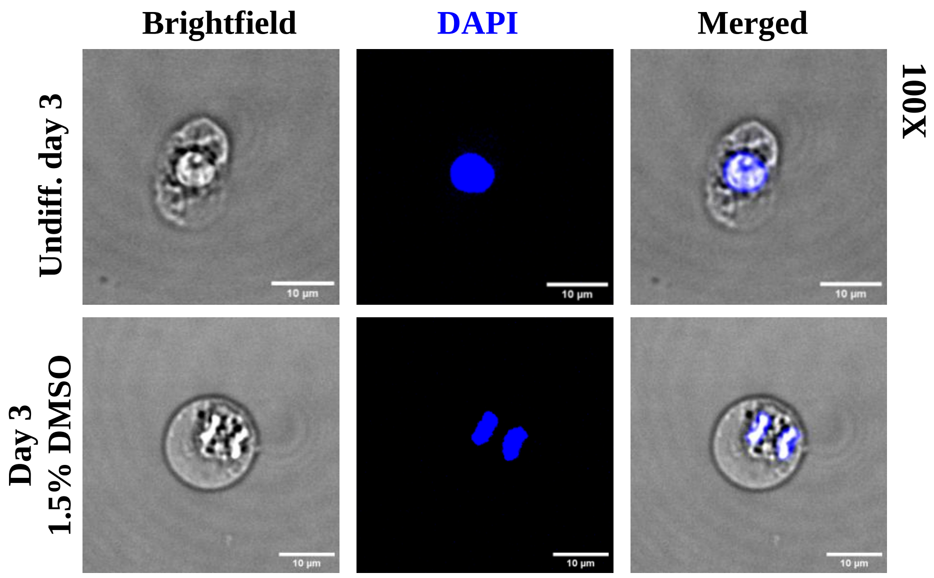

Figure S12. Confocal imaging of HL-60 cells stained with DAPI to visualise nuclear morphology.

Representative confocal micrographs of HL-60 cells showing brightfield images (left), DAPI nuclear staining (middle), and merged overlays (right). The images illustrate the characteristic nuclear morphologies of HL-60 cells, including rounded nuclei in undifferentiated cells and the appearance of multilobed or segmented nuclei, which are typical of differentiated myeloid cells. HL-60 cells have demonstrated nuclear differentiation following treatment with 1.5% DMSO, as evidenced by the presence of prominent multilobed myeloid-type nuclei. Scale bar: 10 µm.

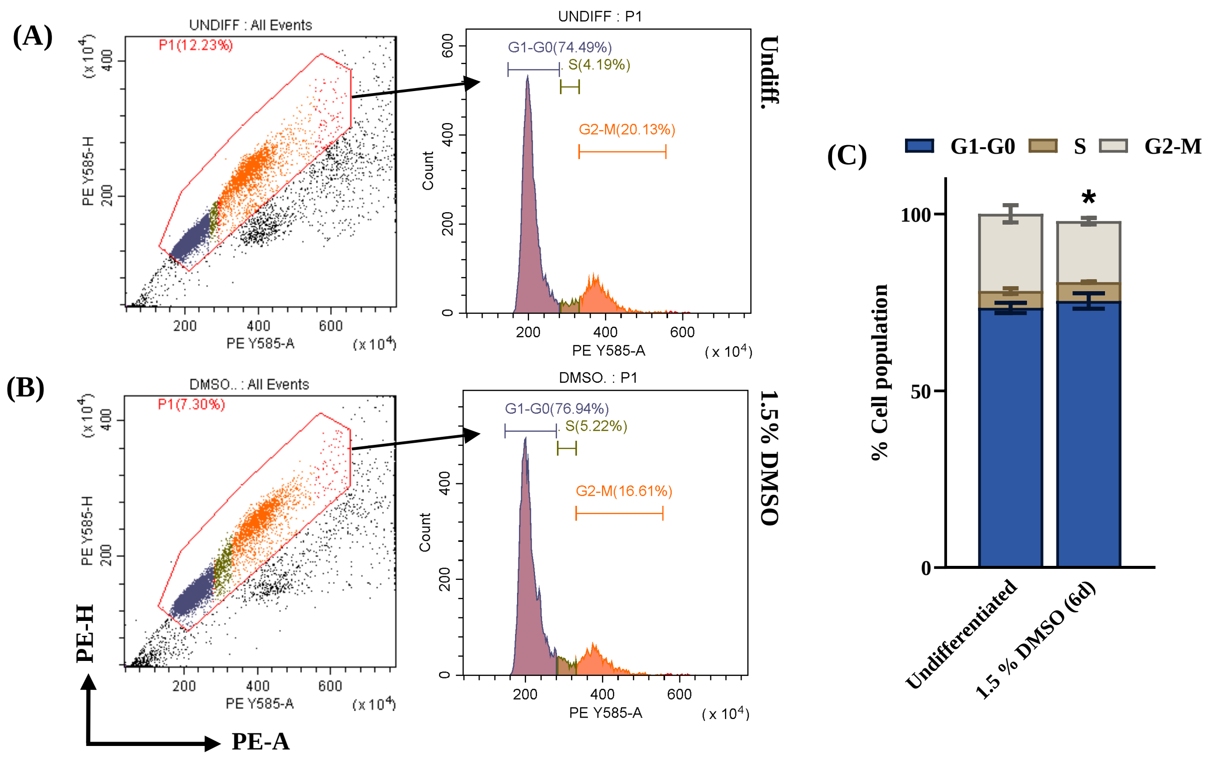

Figure S13. Validation of HL-60 cell differentiation *via* flow cytometry. Cell cycle profiles were determined by propidium iodide (PI) staining and flow cytometric analysis. Representative plots show singlet gating (PE-A *vs*. PE-H) for exclusion of doublets (left panels) and corresponding DNA content histograms (right panels) for (A) Untreated undifferentiated HL60 cells, (B) 1.5% DMSO-treated cells, and. (C) Bar graph depicting the percentage of cells in each phase. Percentages of cells in G_0/G_1, S, and G_2/M phases are indicated in each histogram. A substantial accumulation of cells in the G_0/G_1 phase and a concomitant reduction in G_2/M population were observed, indicating G_0/G_1 cell cycle arrest. Data are presented as the mean ± SD from at least three independent experiments (two-way ANOVA with Tukey's multiple comparisons test, $*P < 0.05$).

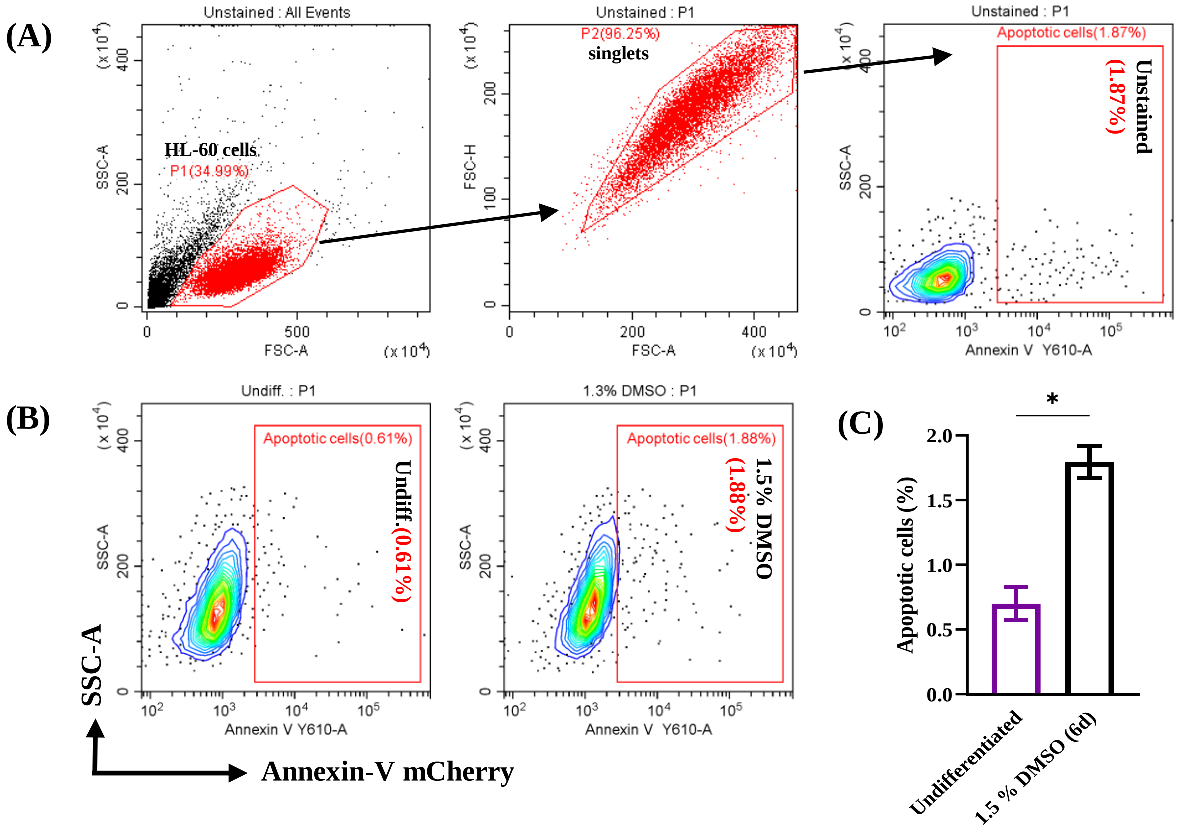

Figure S14. Annexin V–based flow cytometric analysis of apoptosis in HL-60 cells following differentiation. HL-60 cells were analyzed for apoptotic induction using Annexin V-mCherry staining and flow cytometry. The gating strategy included; FSC-A *vs* SSC-A gating to select the main cell population (P1), FSC-A *vs* FSC-H gating to isolate singlets (P2), and Annexin V *vs* SSC plots to quantify apoptotic cells. (A) Gating strategy showing unstained controls used to define the Annexin-V-positive population. (B) Apoptotic percentages in untreated cells and 1.5% DMSO. (C) Bar graphs depicting apoptotic cell percentages in HL-60 cells following differentiation. Only a minimal increase in Annexin-V-positive cells was observed indicating negligible apoptosis under these conditions. Data are presented as the mean ± SD from at least three independent experiments, (two-way ANOVA with Tukey's multiple comparisons test, $^*P < 0.05$).

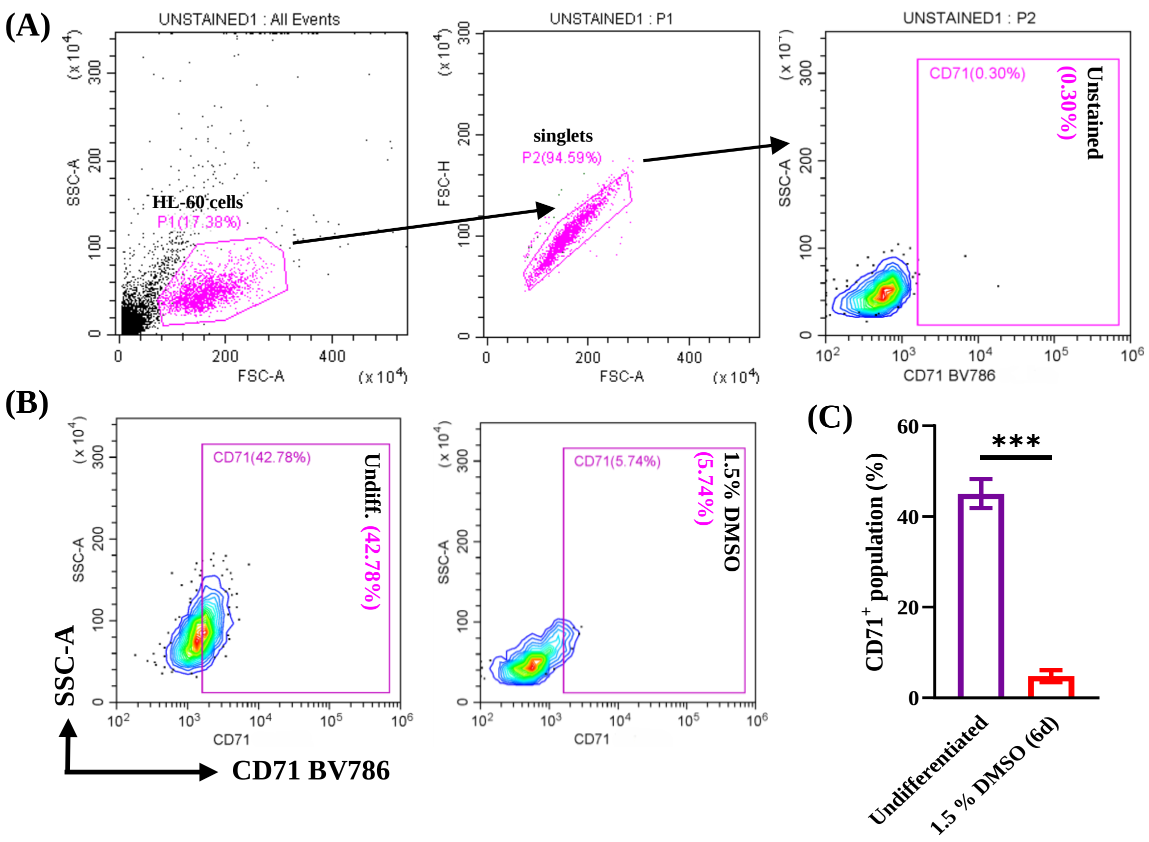

Figure S15. Flow cytometric analysis of CD71 expression during DMSO-induced differentiation in HL-60 cells. HL-60 cells were assessed for surface expression of CD71 (transferrin receptor) following treatment with 1.5% DMSO. The gating strategy included FSC-A *vs* SSC-A selection of the main cell population (P1), followed by FSC-A *vs* FSC-H gating to isolate singlets (P2). (A) CD71 expression was quantified in unstained controls to establish the positive gate. (B) $CD71^+$ populations in untreated cells and 1.5% DMSO-treated cells. (C) Bar graphs depicting $CD71^+$ population in HL-60 cells following differentiation. Untreated control samples showed high CD71 expression (~42–43%; partial differentiation, although not significant), whereas DMSO treatment resulted in a marked reduction of $CD71^+$ cells (~5.7%), ultimately leading to differentiation and downregulation of the transferrin receptor. Data are presented as the mean ± SD from at least three independent experiments (two-way ANOVA with Tukey's multiple comparisons test, ns = not significant, ***$P < 0.01$).

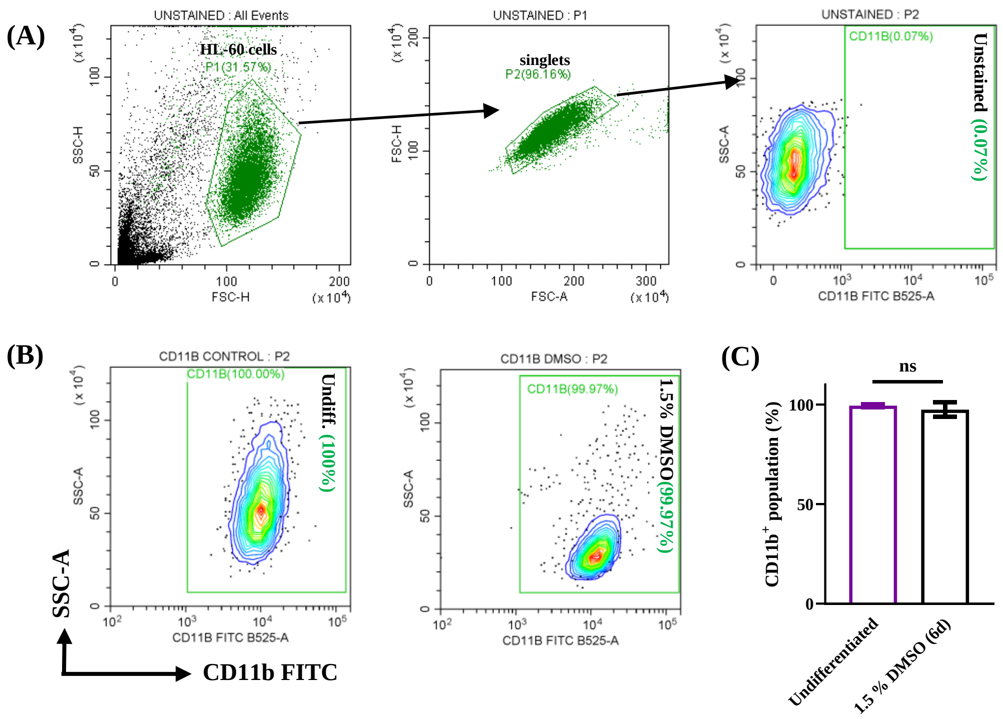

Figure S16. Flow cytometric evaluation of CD11b expression in HL-60 cells following DMSO-induced differentiation. HL-60 cells were analyzed for expression of the myeloid differentiation marker CD11b using FITC-conjugated antibody staining. The gating strategy included FSC-A *vs* SSC-A selection of the main population (P1), followed by FSC-A *vs* FSC-H gating to isolate singlets (P2). (A) Unstained controls were used to define the CD11b-negative region. (B) CD11b expression in untreated HL-60 cells and 1.5% DMSO-treated cells. (C) Bar graphs depicting CD11b$^+$ in HL-60 cells following differentiation. All samples exhibited uniformly high CD11b positivity (~99–100%), consistent with the fact that differentiated HL-60 cells characteristically show near-complete CD11b expression. Data are presented as the mean ± SD from at least three independent experiments (two-way ANOVA with Tukey's multiple comparisons test, ns=not significant).

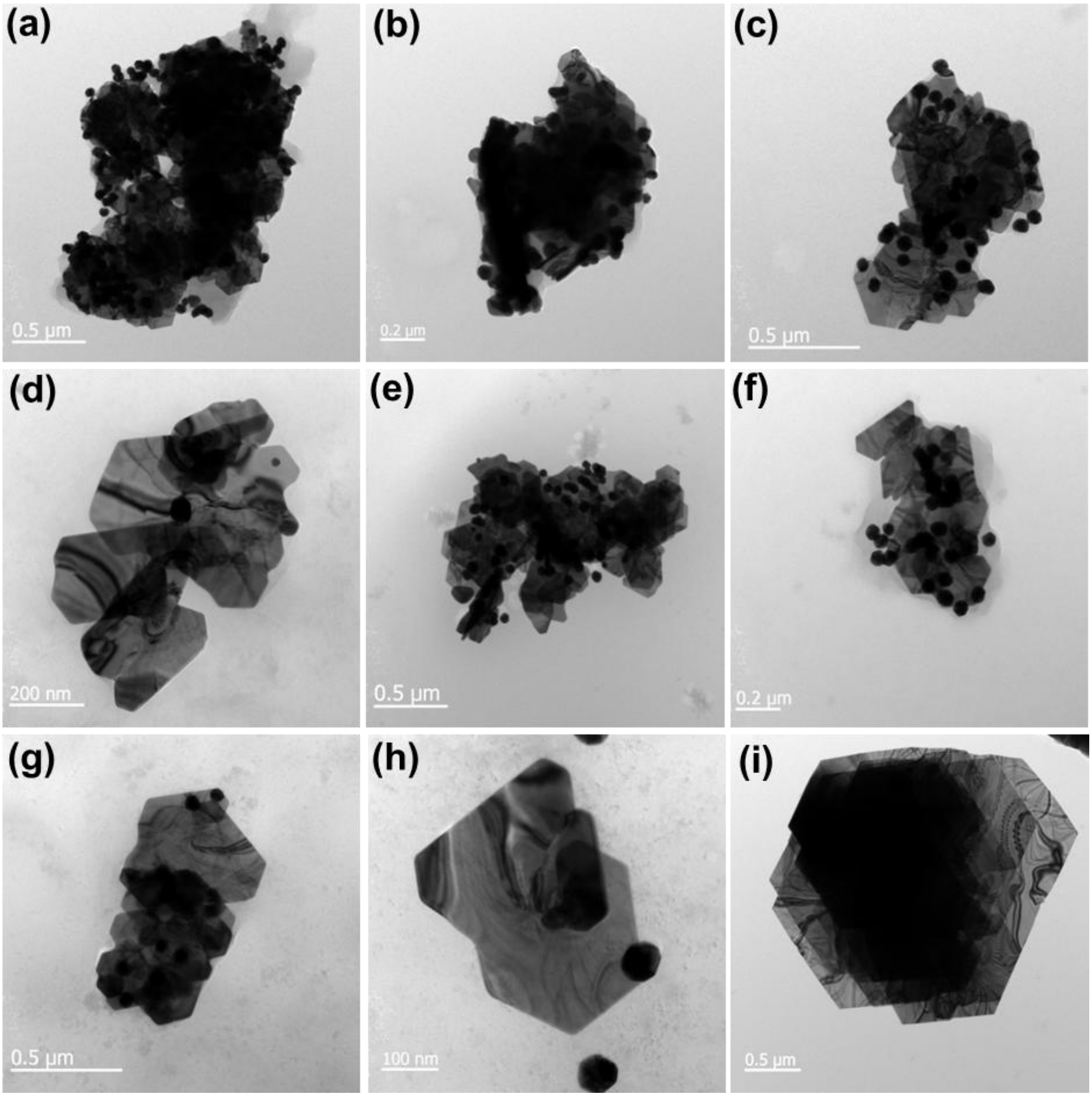

Figure S17. HR-TEM images of in vitro degradation for 14 days; (a-b) AuNS/Cells/Activators, (c-d) AuNS/Cells, (e-f) AuNS/Cells/4-ABAH (20 µM), (g-h) AuNS/Cells/4-ABAH (100 µM), and (i) AuNS/ RPMI Media.

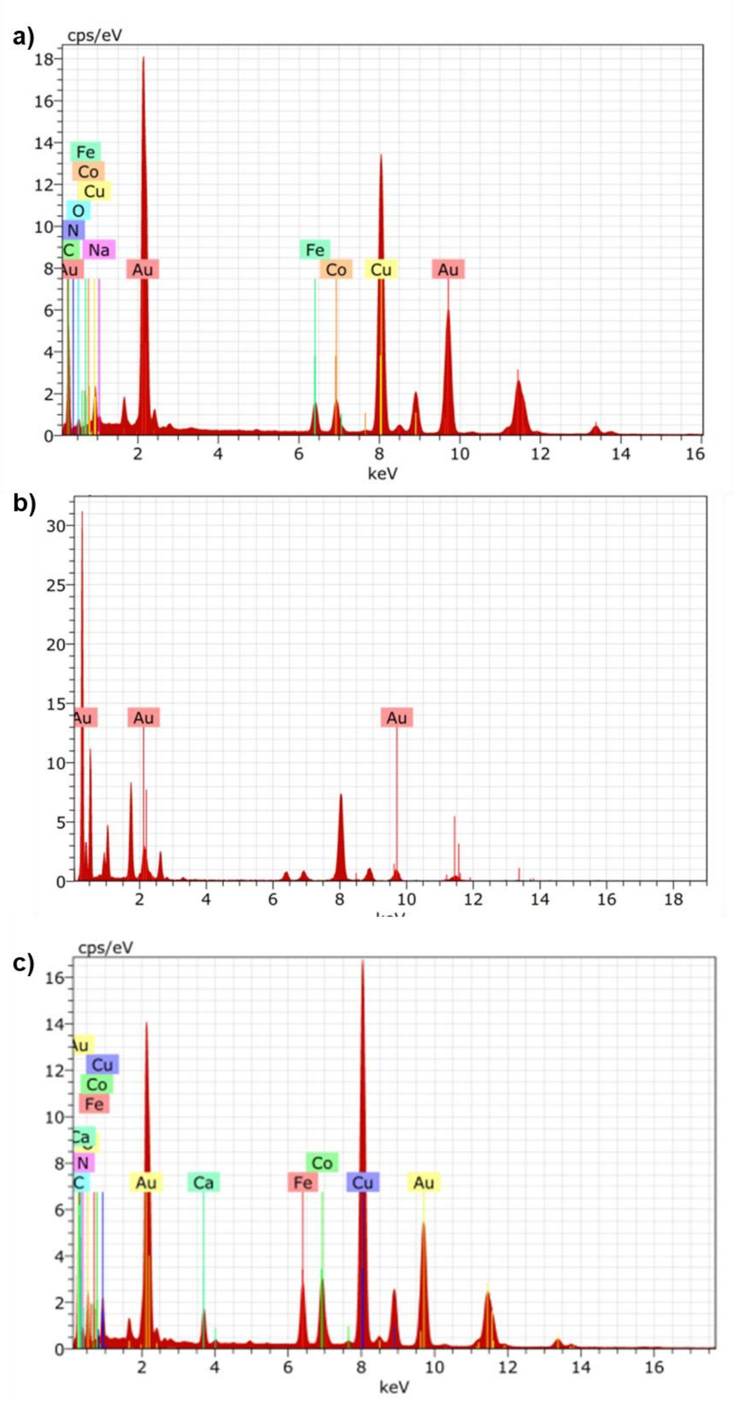

Figure S18. EDS spectrum of a) AuNS (0 day); b) AuNS incubated with neutrophil-like cells (14 days), and c) the spherical structures present in the same sample.

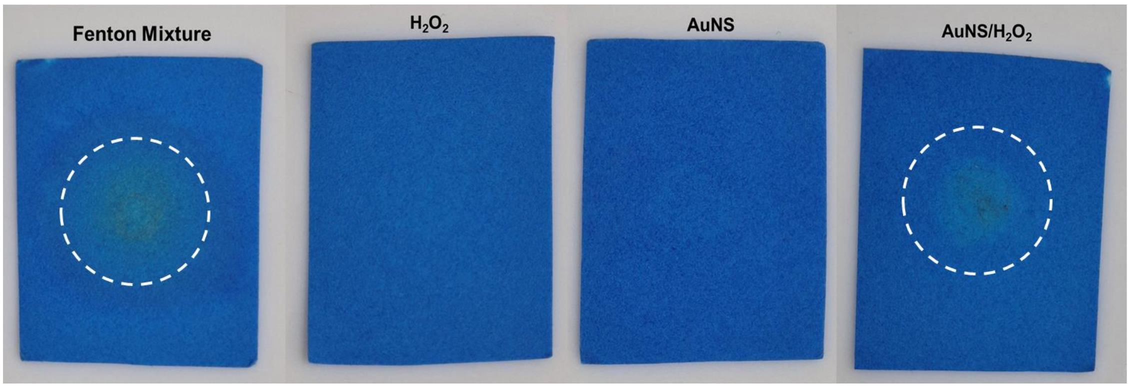

Figure S19: Shows the methylene blue test for generating OH· radicals mediated by AuNS in the presence of H_2O_2, via Fenton-like reaction, catalysed by AuNS.

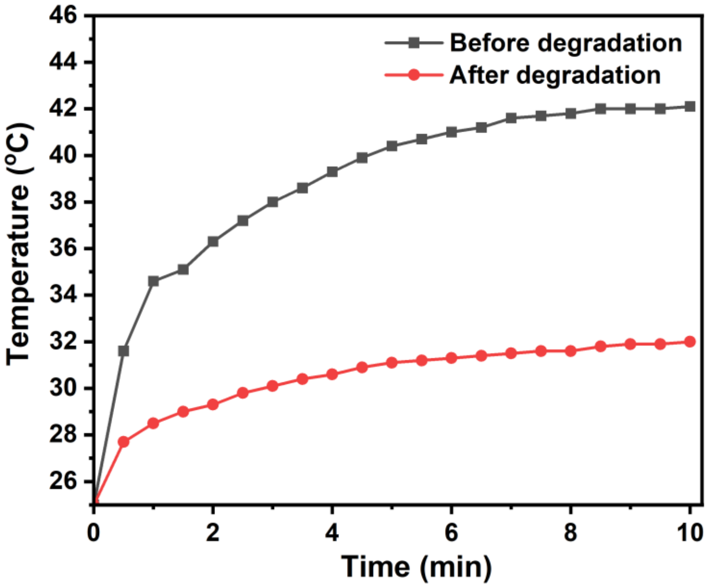

Figure S20. Photothermal temperature rise of AuNS before and after hMPO/H_2O_2 degradation after irradiating with 808 nm NIR laser.

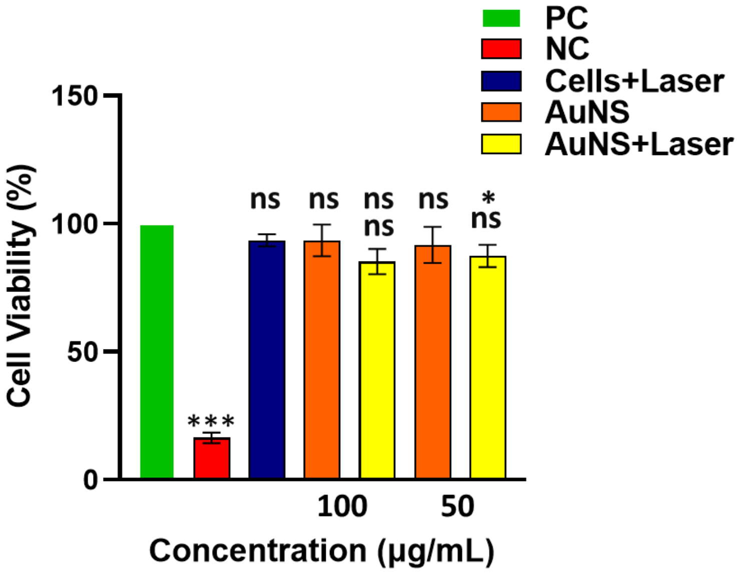

Figure S21. MTT analysis of AuNS after incubating with HEK 293T cells (50 and 100 µL). each concentration vs positive control (above) and laser irradiated sample concentration vs cells + laser (below). PC = positive control, NC = negative control. Statistical significance was calculated using data from experimental replicates using a two-tailed unpaired t-test. *P<0.05, **p<0.01, ***P<0.001, (p)****<0.0001, ns = nonsignificant. Data was represented as mean±SEM
